# miR-150-5p in neutrophil-derived extracellular vesicles associated with sepsis-induced cardiomyopathy in septic patients

**DOI:** 10.1038/s41420-023-01328-x

**Published:** 2023-01-21

**Authors:** Rongzong Ye, Qiuyun Lin, Wenkai Xiao, Lixia Mao, Pengfei Zhang, Lingshan Zhou, Xiaoxia Wu, Nannan Jiang, Xihe Zhang, Yinhua Zhang, Daqing Ma, Jiahao Huang, Xiaoyan Wang, Liehua Deng

**Affiliations:** 1 Department of Critical Care Medicine, Affiliated Hospital of Guangdong Medical University, Zhanjiang, 524000 China; 2 Doctoral Scientific Research Center, Lianjiang People’s Hospital, Zhanjiang, 524400 China; 3 Affiliated Lianjiang People’s Hospital, Guangdong Medical University, Zhanjiang, 524400 China; 4 Laboratory of Southern Marine Science and Engineering, Zhanjiang, 524023 China; 5 Department of Physiology & Biomedical Sciences, Ischemic/hypoxic Disease Institute, Seoul National University College of Medicine, Seoul, 03080 Korea; 6 University Hospital Research Centre, Yanbian University Hospital, Yanji, Jilin Province 133000 China; 7 Division of Anesthetics, Pain, Medicine and Intensive Care, Department of Surgery and Cancer, Faculty of Medicine, Imperial College, London, UK; 8 Chelsea and Westminster, Hospital, London, UK

**Keywords:** Diagnostic markers, Transcriptomics, miRNAs

## Abstract

**Introduction:**

Early diagnosis and potential therapeutic targets of sepsis-induced cardiomyopathy (SIC) remain challenges clinically. Circulating extracellular vesicles from immune cells carrying crucial injurious mediators, including miRNAs in sepsis. However, the impacts of neutrophil-derived extracellular vesicles and their miRNAs in the SIC development are unknown.

**Objectives:**

The present study focused on the in-depth miRNA expression profiles of neutrophil-derived extracellular vesicles and explored the potential molecular biomarkers during the process of SIC.

**Methods:**

Neutrophil-derived extracellular vesicles were isolated from the blood samples in three sepsis patients with or without cardiomyopathy on day 1 and day 3 after ICU admission in comparison with three healthy controls. miRNAs were determined by RNA sequencing. The closely related differentially expressed miRNAs with SIC were further validated through qRT-PCR in the other cohorts of sepsis patients with (30 patients) or without cardiomyopathy (20 patients) and the association between miRNAs and the occurrence or disease severity of septic cardiomyopathy were stratified with logistic regression analysis.

**Results:**

Sixty-eight miRNAs from neutrophil-derived extracellular vesicles were changed significantly between healthy controls and without septic cardiomyopathy patients (61 miRNAs upregulated and seven downregulated). Thirty-eight miRNAs were differentially expressed in the septic cardiomyopathy patients. 27 common differentially expressed miRNAs were found in both groups with similar kinetics (23 miRNAs upregulated and four downregulated). The enriched cellular signaling pathway mediated by miRNAs from sepsis to septic cardiomyopathy was the HIF-1 signaling system modulated septic inflammation. Using multivariate logistic regression analysis, miR-150-5p coupled with NT-pro BNP, LVEF, and SOFA score (AUC = 0.941) were found to be the independent predictors of septic cardiomyopathy.

**Conclusion:**

miRNAs derived from neutrophil-derived extracellular vesicles play an important role in septic disease severity development towards cardiomyopathy. miR-150-5p may be a predictor of sepsis severity development but warrants further study.

## Introduction

Sepsis affects approximately 50 million people worldwide and is a pervasive condition in the intensive care unit (ICU) with high morbidity and mortality. For that reason, the WHO prioritized sepsis among their healthcare policies [1, 2]. Sepsis-induced cardiomyopathy (SIC) is a prevalent complication of sepsis (up to 65%) and it causes exceptionally high death rates [3–5]. It remains a significant challenge to explore new and effective diagnostic and prognostic markers for assessing the status and severity of SIC.

Recently, miRNAs have been identified and validated as biomarkers in various disease types [6]. In sepsis, mounting evidence has implicated miRNAs from immune cells for the early diagnosis of sepsis, as well as predicting the severity and survival of sepsis [7]. However, the miRNA profile is somewhat heterogeneous depending on various studies, e.g., miR-21-3p, miR-146a, and miR-223 [8–10]. It has been acknowledged that human immune cells express cell type-specific [11]. Therefore, it is necessary to specify the types of immune cells for the evaluation of miRNAs as sepsis prognostic biomarkers.

Neutrophils are the most abundant circulating leukocytes, creating the first line of defense against infections caused by invading pathogens. Neutrophils have a vital bearing on immune defense and inflammatory responses, which are involved in the pathogenesis of sepsis-associated acute kidney injury (AKI) [12], acute lung injury (ALI) [13], and acute respiratory distress syndrome (ARDS) [14]. In our previous study [15], we investigated the clinical association between neutrophil-derived microparticles (NDMPs) and sepsis. The results demonstrated that, coupled with pro-inflammatory mediators such as TNF-α, sTREM-1, and IL-6, NDMPs served as a valuable biomarker and could be applied to indicate the severity and mortality of sepsis. In fact, NDMPs are known to express more than 300 signaling molecules [16], demonstrating their impacts on the functions of the targets. Given the significance of the NDMPs, it is plausible that NDMPs can elicit distinct downstream effects and play an important part in the pathogenesis of sepsis. There is thus a paucity of information about the relationship between neutrophil-derived extracellular vesicles and the severity of SIC and we aimed to identify the candidate miRNAs and their impact on patients with SIC.

## Results

### Characterization of neutrophil-derived EVs from septic patients

An overview of the demographic and clinical characteristics of the patients of the two cohorts were presented in Tables 1 and 2. The morphology of EVs, characterized by transmission electron microscopy (TEM), showed that EVs were bowl-shaped with a diameter of about 125 nm (Fig. S1A). To characterize the size distribution of EVs, nanoparticle tracking analysis was conducted, which confirmed that the particle size of EVs varied from 30 to 200 nm (Fig. S1B). In addition, typical markers of EVs, CD9, CD63, and TSG101 were detected in the neutrophil-derived EVs from participants’ peripheral blood samples (Fig. S1C). On the contrary, Calnexin, a negative marker of EVs, was not detected in all the isolated EVs (Fig. S1C). Taken together, these data indicated that neutrophil-derived EVs were consistent with the morphological characteristic of EVs.

**Table 1:** Clinical characteristics of healthy controls and patients with sepsis used for small RNA-sequence analysis of neutrophil-derived EVs.

Variables	Healthy controls	All patients
Number	3	6
Age, years	30 ± 2	70.17 ± 6.210
Male sex, *n*	2	3
**Source of sepsis,** ***n***		
Lung	—	3
Abdominal	—	2
Urinary	—	1
Use of mechanical ventilation, *n* (%)	—	3 (50%)
Duration of mechanical ventilation, days	—	2 (0–12.25)
LVEF D1 (%)	—	54.17 ± 13.060
LVEF D3 (%)	—	53.50 ± 10.055
SOFA score D1	—	16.67 ± 4.502
SOFA score D3	—	16 ± 4.336
APACHE II score D1	—	18 ± 7.294
APACHE II score D3	—	15.67 ± 3.933
ICU length of stay, days	—	6.67 ± 2.944
Hospital length of stay, days	—	11.67 ± 5.955
28-day hospital mortality, n (%)	—	2 (33.33%)

*LVEF* left ventricular ejection fractions, *SOFA* sequential organ failure assessment score, *APACHE* acute physiology and chronic health evaluation, *ICU* intensive care unit.

**Table 2:** The baseline characteristics of all the patients.

Variables	All patients	Non-SIC	SIC	*P*
Number	50	30	20	N/A
Age, years	65 (54–72)	62 (48–71)	67 (63–74)	0.112
Male sex, *n*	38	22	16	0.589
**Comorbidities,** ***n***				
Arterial hypertension	11	7	4	0.780
Diabetes mellitus	11	7	4	0.780
Cerebrovascular disease	4	4	0	0.089
**Source of sepsis,** ***n***				
Lung	26	13	13	0.133
Abdominal	23	14	9	0.908
Skin and soft tissue	6	4	2	0.722
Other	2	1	1	0.768
**Hemodynamic variables**				
MAP, mmHg	68 (61–78)	72 (62–81)	65 (61–70)	0.115
Lactate level, mmol/L	2.47 (1.55–4.34)	2.13 (1.55–3.62)	3 (1.65–4.58)	0.205
**Heart function**				
LVEF (%)	58 (54–60)	59.50 (56–60)	56 (50–60)	0.088
NT-pro BNP, pg/ml	1126 (344–5539)	740.6 (236–1559)	2859.5 (425–9366)	0.072
**Ventilatory data**				
Use of mechanical ventilation, *n* (%)	37	22	15	0.895
PO_2_/FiO_2_, mmHg	248 ± 111	270 ± 121	222 ± 93	0.323
Duration of mechanical ventilation, days	3 (1–7)	2 (0.75–6.25)	4 (0.25–9.50)	0.503
PCT, ng/ml	5.44 (0.54–25.97)	3.58 (0.32–21.98)	10.44 (1.72–45.66)	0.183
WBC,10^12^/L	16.86 (11.83–24.02)	15.69(12.34–21.38)	17.33 (10.85–27.83)	0.496
SOFA score	13.45 ± 4.32	12.23 ± 4.04	15.21 ± 4.22	0.019
APACHE II score	20.5 (17–23)	20 (17–22.25)	22 (17–26)	0.167
ICU length of stay, days	8 (5–11.75)	8 (3.75–11)	8 (5–21)	0.274
Hospital length of stay, days	17 (11–23.75)	12.50 (10.75–20.75)	19 (15–24)	0.159
28-day mortality, *n* (%)	12 (24%)	7 (23.33%)	5 (25%)	0.892

*N/A* not applicable, *MAP* mean arterial pressure, *LVEF* left ventricular ejection fractions, *PO*_*2*_*/FiO*_*2*_ ratio of partial pressure of arterial oxygen to the fraction of inspired oxygen, *PCT* procalcitonin, *WBC* white blood cells, *SOFA* sequential organ failure assessment score, *APACHE* acute physiology and chronic health evaluation, *ICU* intensive care unit.

### miRNA profile of neutrophil-derived EVs were altered significantly in SIC patients

**Fig. 1:**
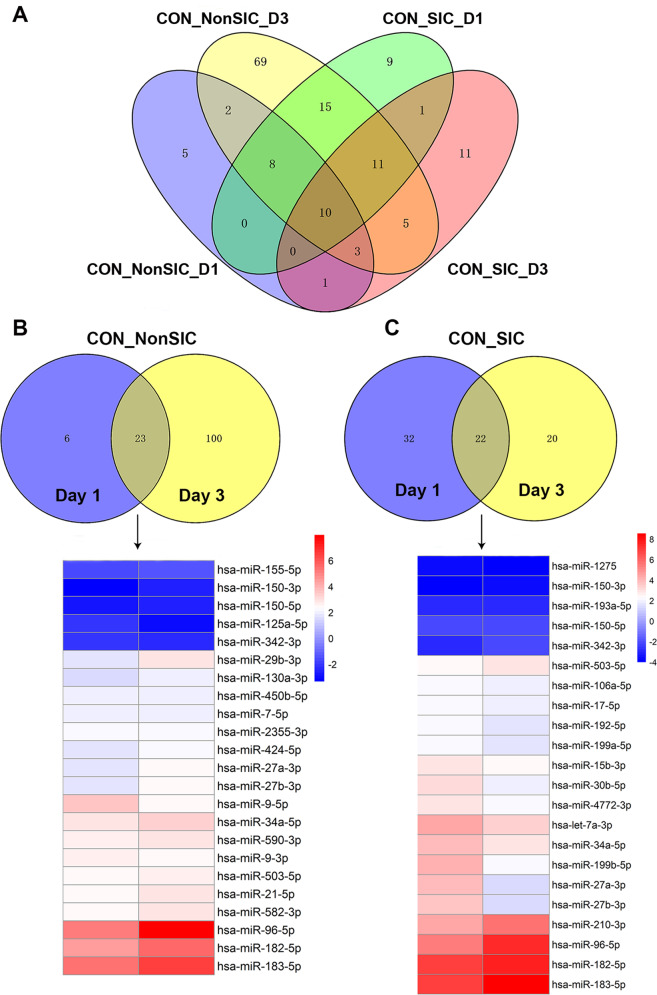
RNA analysis of neutrophil-derived EVs from different participants. **A** Venn diagram illustrating the number of miRNAs from patients with SIC and with non-SIC at ICU admission (D1) and after 3 days (D3) as compared with those of healthy volunteers. **B** The heatmap indicates the fold change profile of the 23 miRNAs differentially expressed at both time points of the non-SIC group versus control subjects. **C** The heatmap indicating the fold change profile of the 22 miRNAs differentially expressed at both time points of the SIC group versus control subjects.

**Fig. 2:**
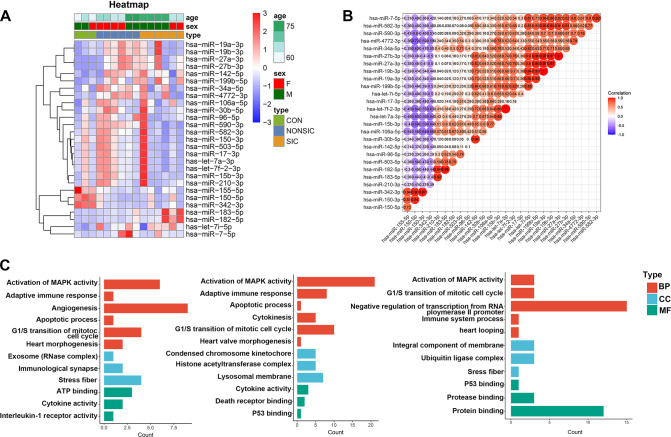
The 27 differentially expressed miRNAs commonly existed in both groups of non-SIC and SIC as compared to the control participants. **A** Hierarchical cluster analysis of the 27 common differentially expressed miRNAs. **B** Correlation matrix for all 27 common differentially expressed miRNAs. Some differentially expressed miRNAs were negatively related (highlighted in blue), and others were positively related (highlighted in red). The darker the color, the higher the correlation was (*p* < 0.05) **C**. GO enmrichment analysis on the three subclasses of protein for the Con vs. NonSIC, Con vs. SIC, NonSIC vs. SIC.

To confirm whether the expression of miRNAs from neutrophil-derived EVs in patients with non-SIC and SIC were different from those of healthy controls, we first performed miRNA profiling analysis in patients with non-SIC and SIC at enrollment and after 3 days as compared with those in healthy individuals (Tables S2,S3). The volcano plot of RNA sequencing data in Fig. S2 graphically depicts the results of differential miRNA expression analysis. Sixty-eight miRNAs from neutrophil-derived extracellular vesicles were changed significantly between healthy controls and without septic cardiomyopathy patients (61 miRNAs upregulated and seven downregulated). Thirty-eight miRNAs were differentially expressed in septic cardiomyopathy patients. We found 40 differently expressed miRNAs in the SIC group compared to the non-SIC group. Twenty-nine miRNAs were differentially expressed in the non-SIC group at enrollment compared with controls. After 3 days, 123 differentially expressed miRNAs were found to be different between the non-SIC group and those of the control group. There were 54 miRNAs in the SIC group and control group at enrollment, and 42 differentially expressed miRNAs were found in the SIC group compared with the controls after 3 days (Fig. 1A). Twenty-three commonly differentially expressed miRNAs found at both time points (D1 and D3) in the non-SIC group showed the same expression kinetics during the disease progression (18 upregulated and five downregulated) (Fig. 1B). Figure 1C demonstrates that these 22 commonly differentially expressed miRNAs at D1 and D3 in SIC group also maintained the same expression trends during SIC course (20 upregulated and two downregulated). Hierarchical clustering was performed for 27 common miRNAs in both non-SIC and SIC groups compared to healthy controls (Fig. 2A). MiR-155-5p, miR-150-5p, and miR-342-3p were observed to be lower in patients with non-SIC and SIC groups when compared to healthy controls. The Corheatmap showed a certain correlation with the percentages of differentially expressed miRNAs (Fig. 2B).

Target genes of differentially expressed miRNAs were further investigated in EVs of SIC, non-SIC, and control groups. The GO analysis of Con vs non-SIC, Con vs SIC, and non-SIC vs SIC is shown in Fig. 2C. Compared to controls, the differentially expressed mRNAs could be predominantly enriched in activation of MAPK activity, cell cycle, and cytokine activity. Such as heart looping and P53 binding focused on the non-SIC vs. SIC. The pathway analysis of the non-SIC group is shown in Figs. S3, S4 illustrate the bioinformatics of our miRNA target database analysis of the SIC group, focusing on the lysosome, hippo signaling pathway, and cell adhesion molecules. A signaling pathway mostly related to p53 was observed in the non-SIC group. Furthermore, NOD-like receptor and Jak-STAT signaling pathways were observed in miRNA enrichment from SIC patients compared to those of control subjects. The target genes of differentially expressed miRNAs were analyzed to determine the processes which may be associated with the KEGG pathway using DIANA-miRPath v.3 platform (Figs. S5, S6) in non-SIC and SIC vs. controls, respectively. The results showed that miR-34a-5p, miR-424-5p, miR-17-5p, and miR-21-5p were associated with a variety of metabolic pathways in two groups. PI3K-Akt signaling pathway and Focal adhesion were downregulated by miR-150-5p in patients with sepsis and SIC. Furthermore, miR-21-5p was shown to have a significant impact on the p53-Akt signaling pathway, cell cycle regulation, and HIF-1 signaling pathway.

### Differential miRNA expression in patients with sepsis and septic shock

According to the sepsis 3.0, we performed a subgroup analysis of the 6 patients in the RNA sequencing into the sepsis (*n* = 3) and septic shock (*n* = 3) groups. The hierarchical clustering analysis (Fig. S7A) displayed the common 23 miRNAs expression levels in the sepsis and septic shock group compared to those of the controls. Enrichment pathway analysis was performed to identify targets for these differentially expressed miRNAs in the sepsis and septic shock groups. These were vastly different when compared to samples from healthy volunteers. Therefore, canonical pathways possibly influenced by these miRNAs were predominantly related to the processes affecting inflammatory responses, such as the NF-κB signaling pathway and TNF signaling pathway (Fig. S7B). A schematic figure of the NF-κB signaling pathway and the TNF signaling pathway are provided in Figs. S8, S9.

### Characterization and verification of candidate miRNAs from neutrophil-derived EVs

**Fig. 3:**
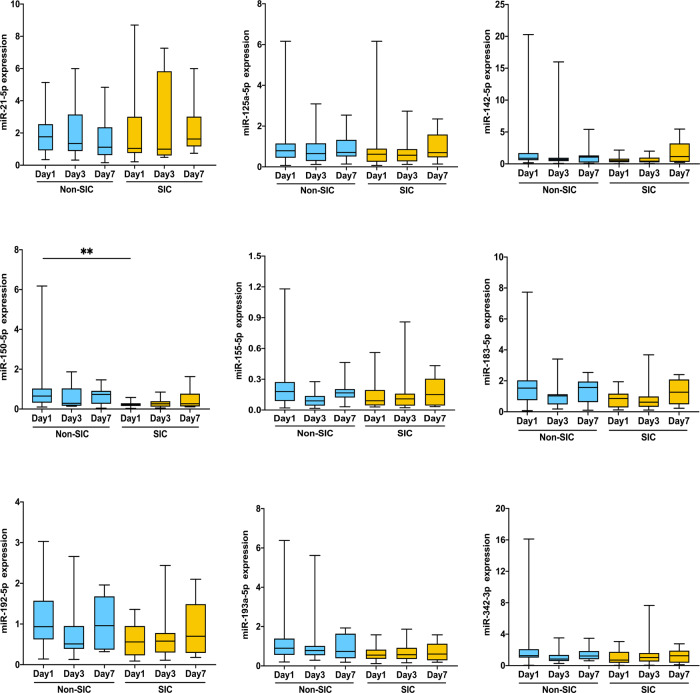
Validation of candidate miRNAs in neutrophil-derived EVs in an independent cohort by qRT-PCR. Non-SIC (*n* = 30) and SIC (*n* = 20); *p < 0.05, **p < 0.001.

Amongst all differentially expressed miRNAs in our study, together with previous literature reports (Table S4), nine miRNAs were qualified between septic patients with (*n* = 20) or without cardiomyopathy (30) and also between healthy controls (*n* = 22) and all septic patients including with or without SIC (*n* = 50). We found that miR-150-5p levels were significantly lower in septic patients with SIC compared to those non-SIC patients (Fig. 3). Patients with SIC also exhibited insignificant trends towards increased expression of miR-21-5p was also higher in SIC patients compared to non-SIC patients but did not reach a statistical significance (Fig. 3). However, when put all septic patients in one pool, miR-21-5p were significantly higher in sepsis patients compared to healthy controls (Fig. S10).

The baseline demographics and laboratory data of liver and kidney function of the validation study population are shown (Table S5). It is noted that there were no significant differences with regard to the liver or renal function between the two groups. To further verify the role of these two miRNAs in the identification of the occurrence of other organ complications in sepsis, the validation cohort was divided into two groups: patients with acute liver failure (ALF) vs Non-ALF or patients with acute kidney injury (AKI) vs non-AKI. However, there were no significant differences with regard to ALF or AKI between the two groups, respectively (Fig. S11A, B). Among Non-SIC subgroup analysis, miR-21-5p and miR-150-5p were not significantly expressed in the septic patients of ALF vs. Non-ALF. Similar results were found in the septic patients of AKI vs. Non-AKI (Fig. S12A, B). Taken together, these findings highlight the specificity of miR-21-5p and miR-150-5p in neutrophil-derived EVs for sepsis and septic cardiomyopathy development, respectively.

### miRNAs in neutrophil-derived EVs as potential biomarkers of SIC

**Fig. 4:**
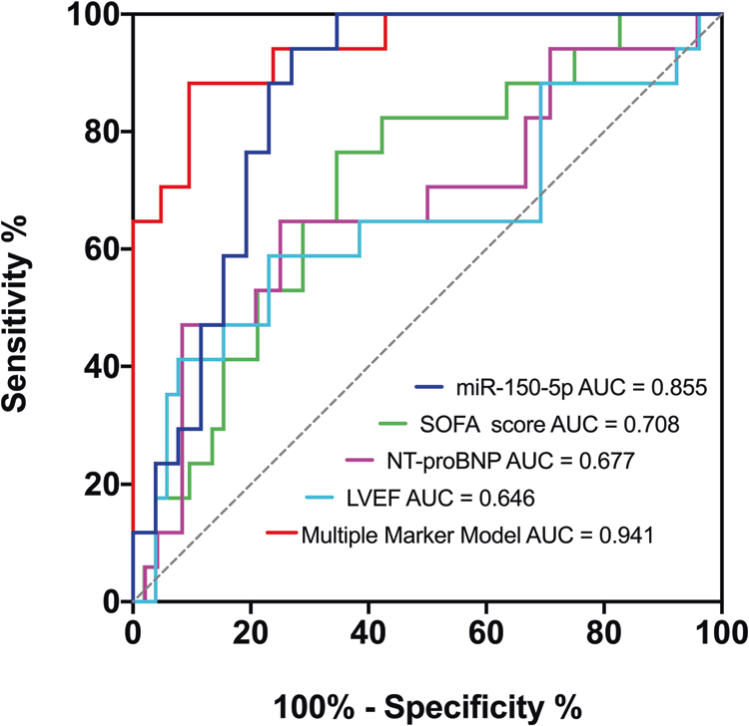
ROC curves for comparing the ability of exosomal miR-150-5p, SOFA score, NT-pro BNP, LVEF, and Multiple Marker Model to discriminate SIC patients from non-SIC. Non-SIC (D1) *n* = 30 biologically independent samples; SIC (D1) *n* = 20 biologically independent samples (validation cohort).

To further assess the predictive value of miRNAs from neutrophil-derived EVs, ROC analysis was performed and the area under the curve (AUC) was calculated. The ROC curves for miR-150-5p and for the multiple marker model (NT-pro BNP, LVEF, and SOFA score) are shown in Fig. 4. ROC curves were then generated for the best single biomarker and the combination model. The AUC for miR-150-5p, NT-pro BNP, LVEF, and SOFA score was 0.941, whereas the AUC for miR-150-5p alone was 0.855. This is advantageous compared to other SIC risk factors, such as NT-pro BNP, LVEF, and SOFA, which achieved the AUC of 0.677, 0.646, and 0.708, respectively (Fig. 4). Taken together, our findings indicate that miR-150-5p in neutrophil-derived EVs could provide promising values to effectively discriminate SIC patients from non-SIC, in addition to the echocardiography and serum cardiac biomarkers.

In multivariate analysis, the logistic regression model was adjusted for age, gender, infection sites, comorbidities, SOFA score, LVEF, NT-pro BNP levels, and miR-150-5p levels. After univariate analysis, only SOFA score, LVEF, NT-pro BNP levels, and miR-150-5p levels were included in the final model. Our results indicated that independent predictors of SIC for septic patients included SOFA (OR = 1.410, 95% CI 1.039–1.914, *p* = 0.028) and miR-150-5p (OR = 0.902, 95% CI 0.842–0.965, *p* = 0.003) (Table 3).

**Table 3:** Multivariable logistic regression of SIC.

Risk Factors	*P*	OR	95%CI	
			Lower	Higher
SOFA	0.028	1.410	1.039	1.914
LVEF	0.094	0.850	0.703	1.028
NT-pro BNP	0.917	1	1	1
miR-150-5p	0.003	0.902	0.842	0.965

*CI* confidence interval, *OR* odds ratio, *SOFA* sequential organ failure assessment score, *LVEF* left ventricular ejection fractions.

**Fig. 5:**
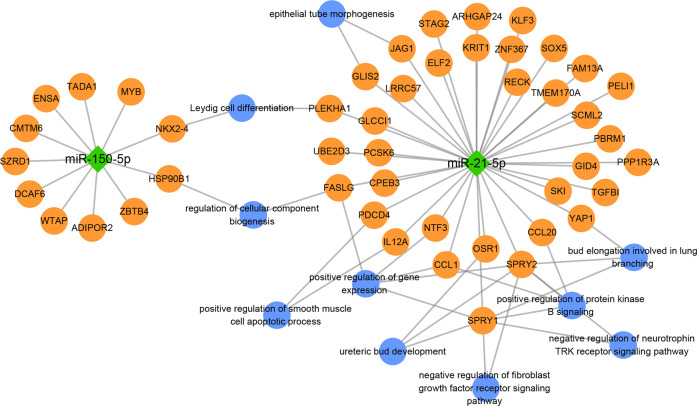
Gene target network of miR-21-5p and miR-150-5p in pathways of relevance to sepsis and SIC. The green diamond represents miRNA; the orange circle represent the target genes of the miRNA; the blue circle represent functional annotations for target genes.

Predicted by the TargetScan, miRanda, and miRDB database, target genes of miR-21-5p and miR-150-5p are both correlated with pathways of potential relevance in sepsis and SIC (Fig. 5). Pathway analysis of these target genes identified a series of canonical biological pathways, which are responsible for cell differentiation, regulation of cellular component biogenesis, gene expression, and cell apoptotic process, as well as signaling pathways related to the regulation of protein kinase B signaling and neurotrophin TPK receptor.

## Discussion

In the current study, the clinical significance of circulating miRNAs from neutrophil-derived extracellular vesicles in a cohort of patients with SIC were systematically analysed and stratified. We demonstrated differences in the regulation of miRNAs during SIC and their targets in the immune system and inflammatory response, which may be responsible for augmenting disease severity. In particular, both miR-21-5p and miR-150-5p were closely related to septic cardiomyopathy. Importantly, miR-150-5p, either alone or together with clinical severity indexes (NT-pro BNP, LVEF, and SOFA score), was able to discriminate patients who might have or may develop SIC with high accuracy. Furthermore, database analyses indicated that the miR-21-5p and miR-150-5p modulated several pathways responsible for sepsis and progression to SIC, mainly including the P53 PI3K-Akt and HIF-1 cellular signaling pathway and cell cycle regulation.

EVs are known to induce peripheral and systemic inflammation during sepsis by carrying contents from activated immune cells [17]. More recently, Vargas et al. [18] reported that intercellular communication was mediated by bioactive neutrophil-derived EVs, raising the possibility of immunoregulatory activities in the pathogenesis of a range of infectious diseases. Very few studies demonstrated the presence of miRNAs in neutrophil-derived EVs during sepsis and the progression to SIC [15]. EVs carried a population of nucleic acids and proteins, and potentially transferred them into the recipient cells via cell-to-cell communication [19]. Previous studies showed that miRNAs are essential regulators that mediate gene expression in a number of physiological and pathophysiological conditions. The interaction events between EVs and the recipient cells promote the exchange of crucial intracellular messages through the release of exosomal molecular cargo, including miRNAs [20–22]. In this regard, our study herein provides key insights into elucidating the connection between exosomal miRNAs of neutrophils and the degree of disease severity during SIC. Through the identification of differentially expressed miRNAs or neutrophil-derived EVs in patients with sepsis and progression to SIC, our data indicate that miRNAs in neutrophil-derived EVs may be as promising circulating biomarkers for diagnostic use for septic patients who have high risk towards SIC prognosis and may facilitate designing new drug design to rein in disease deterioration.

There are several potential mechanisms by which the differential expression of miR-21-5p and miR-150-5p could impact SIC progression. First, miR-150-5p is an evolutionarily conserved miRNA, which has been implicated in a variety of human cancers and inflammation responses that have a close bearing on an unfavorable outcome in patients with critical illness, independent of the presence of sepsis [23]. Cell apoptosis is a crucial mechanism that triggers off pathogenesis of sepsis-induced myocardial depression [24], and indeed, miR-150-5p overexpression alleviated cell apoptosis in rat myocardial tissues and H9c2 cardiomyocytes treated with LPS [25]. Moreover, miR-150 upregulation inhibited TREM-1 expression in splenic conventional dendritic cells and mitigated the inflammatory response in systemic lupus erythematosus [26]. In the present study, we found that the level of miR-150-5p expression in the SIC groups were significantly lower than those in the healthy controls. Thus, the lower level of miR-150-5p in our SIC patients may negate protective mechanisms, including alleviating cell apoptosis and anti-inflammation. In addition, a recent study by Xue et al. suggested that miR-21-5p downregulates the target gene PDCD4 in LPS-treated H9c2 cells and prevents the progression of sepsis [27]. MiR-21-5p was found to be significantly increased in our septic patients compared to healthy controls. All these indicate that it may participate in sepsis development during the disease course. Interestingly, our bioinformatics analyses (Fig. S5) and literature [28] showed that both miR-150-5p and miR-21-5p modulated HIF-1 cellular signaling pathway, whilst the HIF-1α signaling pathway was shown to be attributable to the immunosuppression through the immune cell (e.g., monocytes) phenotype changes reprogramming in the late stage of sepsis.

There are several limitations to this study. For example, this is an exploratory study with a relatively small size of patients. In addition, we excluded patients with chronic diseases, immunosuppression, or tumors. All these may make our conclusions valid in a certain patient population. One can also argue that young healthy individuals were chosen as the normal controls in our study is another limitation. However, the advantage of this age group as control is that there are no comorbidities associated with ageing in relation to miRNA changes per se. On the other hand, our data showed that the neutrophil-derived EVs-associated miR-21-5p and miR-150-5p expressions were not affected by ageing (Fig. S13A, B). Therefore, both miR-150-5p and miR-21-5p from sepsis progression toward sepsis-induced cardiomyopathy should be valid but their biological functions and their cascade are unknown and warrants further study.

## Conclusions

Our study demonstrated the association of neutrophil-derived extracellular vesicles miR-21-5p and miR-150-5p with septic patients and their progression to SIC. Gene set enrichment analysis suggested that miR-150-5p as a negative regulator inhabits the detrimental effects of inflammation and the lower level of miR-150-5p found in our study likely augments disease severity per se. Our study may suggest that neutrophils exosomal miR-150-5p may be used as a predictor of sepsis towards septic cardiomyopathy and/or a therapeutic target for drug treatment development but subjected to further study.

## Materials and methods

### Ethics statement

All experiments involving human patients were conducted according to the ethical policies and procedures approved by the Ethics Committee of the Affiliated Hospital of Guangdong Medical University, China (protocol number: PJ2020-061). Informed consent was obtained from the septic patients and healthy controls in the study.

### Participants

**Fig. 6:**
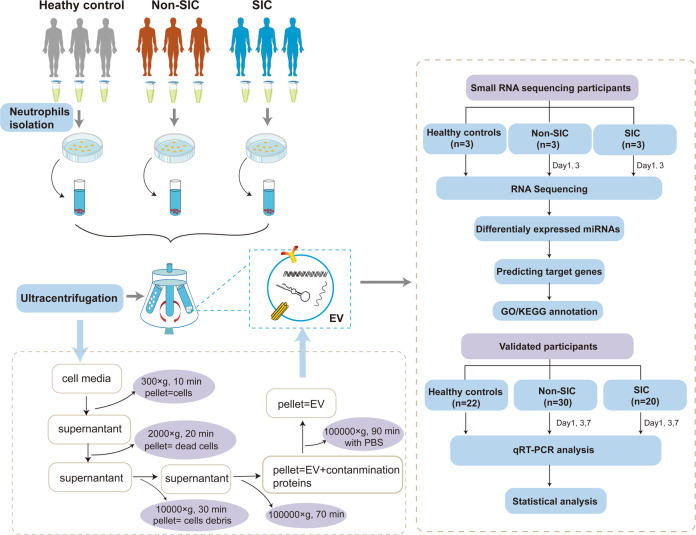
The flow diagram for the experimental procedures. It included the sample collection, sEV extraction, and the small RNA sequencing analysis of sEV from neutrophils in septic patients.

Patients with SIC or sepsis alone (non-SIC) were between 18-85 years old. Sepsis was diagnosed according to the International Sepsis Definition Conference criteria [2]. Routine echocardiography was conducted for all enrolled patients. SIC was defined as follows: a patient with sepsis who was admitted to the ICU and evaluated by the attending physician in combination with relevant clinical indicators, especially a left ventricular ejection fraction (LVEF) of 50% or less on routine echocardiography [4]. We excluded patients who were pregnant, with severe anemia or active bleeding, those with neutropenia, previous history of congenital heart disease, coronary heart disease, myocardial infarction, hypertensive heart disease, pulmonary hypertension, chronic heart dysfunction, tumors or organ transplants, immunosuppressive or immune-deficient state or immunosuppressant medication within the past 6 months or implementation of immunosuppressive therapy such as chemotherapy, and those who failed to complete the study. Twenty-five healthy volunteers were chosen from healthy adults (sex: 11 males and 14 females; ages: 18–50) who had been examined at the medical examination center of the Affiliated Hospital of Guangdong Medical University, China. The workflow of neutrophil-derived exosomal miRNA sequencing was presented in Fig. 6. The first cohort of three healthy volunteers, three SIC, and three non-SIC patients were used to identify altered exosomal miRNAs related to SIC, and the second cohort of 22 healthy volunteers, 20 SIC, and 30 non-SIC patients was used to validate candidates miRNAs related to SIC and study their potential diagnostic use.

### Neutrophil isolation and identification

Blood samples were collected from patients at ICU admission (D1) and 3 days after treatment (D3). Twenty-five peripheral blood was harvested into an EDTA collection tube in the morning and analyzed within 30 min. Human neutrophils from the whole blood samples were isolated with the gradient density centrifugation as reported previously [29]. The resulting cells consisted of more than 85% neutrophils, and the viability of the isolated neutrophils was more than 90% as assessed by flow cytometry and Trypan blue staining, respectively.

### Isolation and characterization of neutrophil-derived EVs

In this study, the pretreatment for removing serum EVs was according to the previous study [30]. Neutrophils were cultured in EVs-free FBS medium for 24 h, after which the culture supernatants were collected and then centrifuged at 300×*g* for 10 min at 4 °C. Neutrophils were removed and supernatants retained and stored in a 50 ml falcon tube at −80 °C until use. Neutrophil-derived EVs were prepared from neutrophil supernatants using differential centrifugation and ultracentrifugation. EVs were visualized using transmission electron microscopy (TEM) as described previously [31]. The size distribution and the total number of EVs were analyzed by nanoparticle tracking analysis with ZetaView PMX 110 (Particle Metrix, Meerbusch, Germany). EV markers such as CD9 (#60232-1, Proteintech), Tsg101(#115706, Abs), CD63 (#sc-5275, Santa), and Calnexin (#10427-2, Proteintech) were determined by Western blot.

### RNA extraction and RNA sequencing

Total RNA was extracted and purified from neutrophil-derived extracellular vesicles using a miRNeasy® Mini kit (Qiagen, Valencia, CA, USA) accordingly. Total RNA from the first cohort of three healthy volunteers, three SIC, and three non-SIC patients was isolated and then used for RNA sequencing. The RNA sequencing libraries (Echo Biotech, Beijing, China) were prepared for analysis and RNA sequencing was conducted with an Illumina Novaseq 6000 platform and 150 bp-paired end reads.

### Quantification and differential expression analysis of miRNA

Quantification and differential expression analysis of miRNA were performed as previously described [32].

### Target gene prediction and GO/KEGG pathway enrichment analysis

For each miRNA with differential expression between healthy controls and sepsis, its potential target genes predicted by DIANA-miRPath, TarBase, microT-CDS, and TargetScan were included for further analysis. Then, Blast was used to compare the target gene sequences with known sequences in Gene Ontology (GO) and Kyoto Encyclopedia of Genes and Genomes (KEGG) databases to determine the potential biological functions of the target genes. GOseq R packages based on Wallenius non-central hypergeometric distribution were used for GO enrichment analysis. KEGG pathway enrichment was analyzed by the python program KOBAS, as described in a previous study [33].

### Quantitative real-time PCR

To validate miRNAs identified in RNA sequencing data, we performed qRT-PCR analysis for the selected miRNA targets. Total RNA was extracted and purified from neutrophil-derived EVs using miRNeasy® Mini kits (Qiagen, Valencia, CA, USA). 50 ng of total RNA from each sample underwent reverse transcription into the corresponding cDNA by using the Mir-X^TM^ miRNA First-Strand Synthesis Kit (TaKaRa Biotechnology, USA). Real-time quantitative PCR was performed with miRNA-specific primer pairs (Table S1) (Sangon Biotech, Shanghai, China) by using the TB Green Advantage qPCR Premix (TaKaRa Biotechnology, USA). We used U6 as an endogenous control miRNA when determining miRNA levels in neutrophil-derived EVs. The cycle thresholds (Ct) for miRNAs and the reference miRNA from each individual sample were determined. For calculation, ΔCt = Ct_miRNA_ – Ct_reference gene_ and ΔΔCt = ΔCt_patient_ – ΔCt_control_. The fold of miRNA expression in the patient group over the control group is 2^−ΔΔCt^, as described in a previous study [34].

### Statistical analysis

RNA sequencing data analyses were conducted with the statistical R package. All variables were assessed for normality and analysis of variance (ANOVA) for continuous variables and the chi-squared test for categorical variables. Comparisons of continuous normal distribution variables were performed using student tests and presented as mean (SD), while comparisons of nonnormal distribution variables were performed using Wilcoxon rank-sum tests and presented as median (interquartile range [IQR]). Receiver operating characteristic (ROC) curves analysis and Logistic Regression were applied to evaluate the efficiency of diagnostic and/or prognosis parameters. All analyses were performed with GraphPad Prism 9.0.2 and SPSS 26.0. A value of *P* < 0.05 was considered to be of statistical significance.

## Supplementary information

Supplemental Figure legends

Table S1

Table S2

Table S3

Table S4

Table S5

Figure S1

Figure S2

Figure S3

Figure S4

Figure S5

Figure S6

Figure S7

Figure S8

Figure S9

Figure S10

Figure S11

Figure S12

Figure S13

Original Data File

Original Data File

Original Data File

Original Data File

## Data Availability

The data that support this study are available from the corresponding authors upon reasonable request.
