# Supplementary material for: miR-150-5p in neutrophil-derived extracellular vesicles associated with sepsis-induced cardiomyopathy in septic patients
Source: Cell Death Discov. 2023 Jan 21;9:19. doi: 10.1038/s41420-023-01328-x (PMC9867758; doi:10.1038/s41420-023-01328-x)
Supplement: Supplementary file 1 — Supplemental Figure legends [file 41420_2023_1328_MOESM1_ESM.docx]

**Fig. S1.** Isolation and characterization of neutrophil-derived EVs from sepsis with SIC and NonSIC. **A**. Representative transmission electron microscopy (TEM) images showed that neutrophil-derived EVs were oval or bowl-shaped capsules without the nucleus. **B**. Nanoparticle tracking analysis results suggested that neutrophil-derived EVs enriched from circulating were about 100-200 nm in diameter. **C**. EVs markers CD9, TSG101 and CD63, were all detected in the neutrophil-derived EVs isolated from the peripheral blood, and Calnexin, a negative marker of EVs was absent in our isolated neutrophil-derived EVs.

**Fig. S2.** Volcano plot of RNA sequencing data. **A**. Controls vs Non-SIC **B**. Controls vs SIC **C**. Non-SIC vs SIC.Red: miRNA strongly up-regulated (FC>1.5, FDR<0.05); Blue: miRNA deregulated (FC>1.5, FDR<0.05). Grey: no significantly expressed miRNAs.

**Fig. S3.** KEGG classification diagram of NonSIC group. The ordinate is the name of the KEGG metabolic pathway, and the abscissa is the ratio of the number of genes annotated to the pathway and the total number of genes annotated. Genes are divided into six branches based on KEGG metabolic pathways (genetic information processing; organismal systems; cellular processes; environmental information processing; human disease; metabolism)

**Fig. S4.** KEGG classification diagram of SIC group. The ordinate is the name of the KEGG metabolic pathway, and the abscissa is the ratio of the number of genes annotated to the pathway and the total number of genes annotated. Genes are divided into six branches based on KEGG metabolic pathways (genetic information processing; organismal systems; cellular processes; environmental information processing; human disease; metabolism)

**Fig. S5.** Pathway analysis of differentially expressed miRNAs using DIANA miRPath v.3. Pathway analysis for differentially expressed miRNAs in NonSIC group.

**Fig. S6.** Pathway analysis of differentially expressed miRNAs using DIANA miRPath v.3. Pathway analysis for differentially expressed miRNAs in SIC group.

**Fig. S7.** The 23 common differentially expressed miRNAs and the corresponding target genes were associated with KEGG in sepsis and septic shock compared to the controls. **A.** Pattens of miRNA expression from EVs of patients with sepsis (S) and septic shock (SS), compared to the healthy control subjects. The figure shows the analysis by hierarchical clustering of samples considering the change in cycle threshold values after filtering and normalization of data. **B.** Biological pathways potentially influenced by differentially expressed miRNAs carried by neutrophil-derived EVs of patients with sepsis and septic shock compared with those of healthy individuals. C: healthy individuals; S: sepsis; SS: septic shock; M: male; F: female.

**Fig. S8.** Targets of miRNAs in neutrophil-derived EVs expressed in sepsis involved in the NF-κB signaling pathway. Red colors represent up-regulated target gene.

**Fig. S9.** Targets of Targets of miRNAs in neutrophil-derived EVs expressed in sepsis involved in the TNF signaling pathway. Red colors represent down-regulated target gene.
**Fig. S10.** Validation of candidate miRNAs in neutrophil-derived EVs in subgroup cohort by qRT-PCR. Health control (n=22) and Sepsis (n=50); *p < 0.05, **p < 0.001, ***p < 0.0001.

**Fig. S11.** Investigation of SIC-related miRNAs in an independent cohort by qRT-PCR. A. Expression of neutrophil-derived EVs-associated miR-21-5p and miR-150-5p was analyzed in 2 groups of sepsis patients with ALF and without ALF. n=34 for Non-ALF sepsis group, n=16 for ALF sepsis group; B. Expression of neutrophil-derived EVs-associated miR-21-5p and miR-150-5p was analyzed in 2 groups of sepsis patients with AKI and without AKI. n=22 for Non-AKI sepsis group, n=28 for AKI sepsis group. U6 was used as an endogenous control. Data are expressed as the mean ± SEM. *P < 0.05.

**Fig. S12.** Investigation of SIC-related miRNAs in a Non-SIC Subgroup analysis by qRT-PCR. A. Expression of neutrophil-derived EVs-associated miR-21-5p and miR-150-5p was analyzed in 2 groups of sepsis patients with ALF and without ALF. n=23 for Non-ALF sepsis group, n=7 for ALF sepsis group. B. Expression of neutrophil-derived EVs-associated miR-21-5p and miR-150-5p was analyzed in 2 groups of sepsis patients with AKI and without AKI. n=19 for Non-AKI sepsis group, n=11 for AKI sepsis group. U6 was used as an endogenous control. Data are expressed as the mean ± SEM. *P < 0.05.

**Fig. S13**. Investigation of neutrophil-derived EVs-associated miR-21-5p and miR-150-5p from patients with age. A. Expression of neutrophil-derived EVs-associated miR-21-5p and miR-150-5p was analyzed in 2 groups of sepsis patients with age by qRT-PCR. n = 18 for age≤60 years, n = 32 for age>60 years; *P < 0.05 significant compared with control group. B. Spearman correlation analysis of miR-21-5p and miR-150-5p expression with ageing.
