# Supplementary material for: miR-150-5p in neutrophil-derived extracellular vesicles associated with sepsis-induced cardiomyopathy in septic patients
Source: Cell Death Discov. 2023 Jan 21;9:19. doi: 10.1038/s41420-023-01328-x (PMC9867758; doi:10.1038/s41420-023-01328-x)
Supplement: Supplementary file 2 — Table S1 [file 41420_2023_1328_MOESM2_ESM.docx]

| **miRNA ID** | **Forward** |
| --- | --- |
| miR-21-5p | TAGCTTATCAGACTGATGTTGA |
| miR-125a-5p | TCCCTGAGACCCTTTAACCTGTGA |
| miR-142-5p | CATAAAGTAGAAAGCACTACT |
| miR-150-5p | TCTCCCAACCCTTGTACCAGTG |
| miR-155-5p | TTAATGCTAATCGTGATAGGGGTT |
| miR-183-5p | TATGGCACTGGTAGAATTCACT |
| miR-192-5p | CTGACCTATGAATTGACAGCC |
| miR-193-5p | TGGGTCTTTGCGGGCGAGATGA |
| miR-342-3p | TCTCACACAGAAATCGCACCCGT |

**Table S1** The primers of selected differently expressed miRNA candidates
