# Supplementary material for: miR-150-5p in neutrophil-derived extracellular vesicles associated with sepsis-induced cardiomyopathy in septic patients
Source: Cell Death Discov. 2023 Jan 21;9:19. doi: 10.1038/s41420-023-01328-x (PMC9867758; doi:10.1038/s41420-023-01328-x)
Supplement: Supplementary file 5 — Table S4 [file 41420_2023_1328_MOESM5_ESM.docx]

**Table S4** Information of the Selected miRNAs for RT-qPCR Verification

| **miR_ID** | **Reported functioning in sepsis** |
| --- | --- |
| miR-21-5p | miR-21-5p dysregulation in exosomes derived from heart failure patients impairs regenerative potentia^[1]^. miR-21-5p plays a key role in hMSC-exo-mediated effects on cardiac contractility and calcium handling, likely via PI3K signaling^[2]^. |
| miR-125a-5p | miR-125a-5p altered in heart failure patients when compared with those without clinical events^[3]^. Long noncoding RNA NEAT1 sponges miR‑125a‑5p to suppress cardiomyocyte apoptosis via BCL2L12^[4]^. |
| miR-142-5p | miR-142 could attenuate CLP-induced inflammation and thus sepsis via targeting PD-L1 in macrophages ^[5]^. |
| miR-150-5p | miR-150-5p down regulated, its overexpression attenuates myocardial apoptosis ^[6]^. The lncRNA XIST/miR-150-5p/c-Fos axis regulates sepsis-induced myocardial injury via TXNIP-modulated pyroptosis^[7]^. |
| miR-155-5p | miR-155 overexpression attenuates myocardial damage ^[8]^. miR-155-5p, may be involved in the regulation of endothelial permeability ^[9]^. |
| miR-183-5p | In vitro and in vivo study revealed that miR-183 improves myocardial damager through the NF-κB pathway ^[10]^. Hemin-MSC-Exosomal miR-183-5p mediates the cardioprotective effects of Hemin-MSC-EXO by inhibiting cardiomyocyte senescence via regulation of the HMGB1/ERK pathway ^[11]^. |
| miR-192-5p | LncRNA KCNQ1OT1 attenuates sepsis-induced myocardial injury via regulating miR-192-5p/XIAP axis^[12]^. |
| miR-193a-5p | Differentiated patients with an infectious disease (CAP or sepsis) from volunteers ^[13]^. Protecting endothelial cells from oxidative stress damage via ACVR1 ^[14]^. |
| miR-342-3p | Targets several proteins in LPS signaling pathway ^[15]^. Plasma exosomal miR-342-3p contributed to exosome-mediated heart repair by inhibiting cardiomyocyte apoptosis and autophagy through targeting SOX6 and TFEB, respectively^[16]^. |

**Abreviations:** ACVR1= activin a receptor type 1; BCL2L12= B cell leukemia 2-like 12

CLP= cecal ligation and puncture; CAP= community-acquired pneumonia; EXO= exosome; HMGB1= high mobility group box protein 1; LPS= lipopolysaccharide; MSC= mesenchymal stromal cell; NF-κB= nuclear factor kappa B; PD-L1= programmed death-ligand 1;

**References**

Qiao L, Hu S, Liu S, Zhang H, Ma H, Huang K, Li Z, Su T, Vandergriff A, Tang J, Allen T, Dinh PU, Cores J, et al. microRNA-21-5p dysregulation in exosomes derived from heart failure patients impairs regenerative potential. J Clin Invest. 2019; 129(6):2237-2250.

Mayourian J, Ceholski DK, Gorski PA, Mathiyalagan P, Murphy JF, Salazar SI, Stillitano F, Hare JM, Sahoo S, Hajjar RJ, Costa KD. Exosomal microRNA-21-5p Mediates Mesenchymal Stem Cell Paracrine Effects on Human Cardiac Tissue Contractility. Circ Res 2018 03 30;122(7).

Galluzzo A, Gallo S, Pardini B, Birolo G, Fariselli P, Boretto P, Vitacolonna A, Peraldo-Neia C, Spilinga M, Volpe A, Celentani D, Pidello S, Bonzano A, Matullo G, Giustetto C, Bergerone S, Crepaldi T. Identification of novel circulating microRNAs in advanced heart failure by next-generation sequencing. ESC Heart Fail 2021 08;8(4).

Yan H, Liang H, Liu L, Chen D and Zhang Q. Long noncoding RNA NEAT1 sponges miR125a5p to suppress cardiomyocyte apoptosis via BCL2L12. Mol Med Rep. 2019; 19(5):4468-4474.

Zhen J and Chen W. MiR-142 inhibits cecal ligation and puncture (CLP)-induced inflammation via inhibiting PD-L1 expression in macrophages and improves survival in septic mice. Biomed Pharmacother. 2018; 97:1479-1485.

Zhu XG, Zhang TN, Wen R and Liu CF. Overexpression of miR-150-5p Alleviates Apoptosis in Sepsis-Induced Myocardial Depression. Biomed Res Int. 2020; 2020:3023186.

Wang X,  Li XL,  Qin LJ. The lncRNA XIST/miR-150-5p/c-Fos axis regulates sepsis-induced myocardial injury via TXNIP-modulated pyroptosis. Lab Invest 2021 09;101(9).

Zhou Y, Song Y, Shaikh Z, Li H, Zhang H, Caudle Y, Zheng S, Yan H, Hu D, Stuart C and Yin D. MicroRNA-155 attenuates late sepsis-induced cardiac dysfunction through JNK and beta-arrestin 2. Oncotarget. 2017; 8(29):47317-47329.

Maucher D, Schmidt B, Schumann J. Loss of Endothelial Barrier Function in the Inflammatory Setting: Indication for a Cytokine-Mediated Post-Transcriptional Mechanism by Virtue of Upregulation of miRNAs miR-29a-3p, miR-29b-3p, and miR-155-5p. Cells 2021 10 22;10(11) .

Xing J, Xie T, Tan W, Li R, Yu C and Han X. microRNA-183 improve myocardial damager via NF-kb pathway: In vitro and in vivo study. J Cell Biochem. 2019; 120(6):10145-10154.

Zheng H, Liang X, Han Q, Shao Z, Zhang Y, Shi L, Hong Y, Li W, Mai C, Mo Q, Fu Q, Ma X, Lin F, Li M, Hu B, Li X, Zhang Y. Hemin enhances the cardioprotective effects of mesenchymal stem cell-derived exosomes against infarction via amelioration of cardiomyocyte senescence. J Nanobiotechnology 2021 Oct 21;19(1).

Sun F, Yuan W, Wu H, Chen G, Sun Y, Yuan L, Zhang W and Lei M. LncRNA KCNQ1OT1 attenuates sepsis-induced myocardial injury via regulating miR-192-5p/XIAP axis. Exp Biol Med (Maywood). 2020; 245(7):620-630.

Hermann S, Brandes F, Kirchner B, Buschmann D, Borrmann M, Klein M, Kotschote S, Bonin M, Reithmair M, Kaufmann I, Schelling G and Pfaffl MW. Diagnostic potential of circulating cell-free microRNAs for community-acquired pneumonia and pneumonia-related sepsis. J Cell Mol Med. 2020; 24(20):12054-12064.

Cao C, Wang B, Tang J, Zhao J, Guo J, Guo Q, Yue X, Zhang Z, Liu G, Zhang H, Wang Y and Zhang J. Circulating exosomes repair endothelial cell damage by delivering miR-193a-5p. J Cell Mol Med. 2021; 25(4):2176-2189.

Schmidt WM, Spiel AO, Jilma B, Wolzt M and Muller M. In vivo profile of the human leukocyte microRNA response to endotoxemia. Biochem Biophys Res Commun. 2009; 380(3):437-441.

Wang B, Cao C, Han D, Bai J, Guo J, Guo Q, Li D, Zhang J, Zhang Z, Wang Y, Tang J, Shen D, Zhang J. Dysregulation of miR-342-3p in plasma exosomes derived from convalescent AMI patients and its consequences on cardiac repair. Biomed Pharmacother 2021 Oct;142.
