# Supplementary material for: miR-150-5p in neutrophil-derived extracellular vesicles associated with sepsis-induced cardiomyopathy in septic patients
Source: Cell Death Discov. 2023 Jan 21;9:19. doi: 10.1038/s41420-023-01328-x (PMC9867758; doi:10.1038/s41420-023-01328-x)
Supplement: Supplementary file 6 — Table S5 [file 41420_2023_1328_MOESM6_ESM.docx]

**Table S5** The baseline characteristics of all the patients

| **Variables** | **All patients** | **Non-SIC** | **SIC** | ***P*** |
| --- | --- | --- | --- | --- |
| Number | 50 | 30 | 20 | N/A |
| Age, years | 65 (54-72） | 62 (48-71） | 67 (63-74） | 0.112 |
| Male sex, n | 38 | 22 | 16 | 0.589 |
| **Liver function** |  |  |  |  |
| PT-TIME, s | 15.10 (13.05-19.05) | 14.70 (12.80-16.45) | 18.05 (13.40-19.63) | 0.097 |
| APTT-TIME, s | 42.50 (33.65-48.25) | 39.80 (29.25-46.75) | 44.30 (37.95-57.40) | 0.017 |
| FIB, g/L | 4.87 ± 2.16 | 4.74 ± 2.60 | 5.07 ± 1.28 | 0.155 |
| ALT, U/L | 27.40 (14.20-53.05) | 25.70 (14.20-42.95) | 39.20  (10.83-152) | 0.246 |
| AST, U/L | 34.30 (20.65-104.90) | 27.80 (19.50-61.95) | 79.10 (33.28-219.43) | 0.055 |
| TB, μmol/L | 12.60 (8.05-27.60) | 12.40  (7.70-19.25) | 21.80 (10.08-46.43) | 0.174 |
| ALB, g/L | 28.61 ± 5.38 | 29.59 ± 5.85 | 27.07 ± 4.27 | 0.146 |
| **Renal function** |  |  |  |  |
| Scr, μmol/L | 87 (70.50-165.50) | 82 (65.50-118) | 104.50 (71-277.25) | 0.322 |
| CysC, mg/L | 1.2 (0.89-1.46) | 1.14 (0.86-1.53) | 1.21 (1-1.40) | 0.641 |

**Abreviations:** N/A= not applicable; APTT= activated plasma thromboplastin time；ALT= alanine aminotransferase；AST= aspartate aminotransferase; ALB= albumin; CysC= cystatin C ; FIB= fibrinogen; PT= prothrombin time; Scr= serum creatinine ; TB = total bilirubin.
