# Supplementary figures and images for: miR-150-5p in neutrophil-derived extracellular vesicles associated with sepsis-induced cardiomyopathy in septic patients
Source: Cell Death Discov. 2023 Jan 21;9:19. doi: 10.1038/s41420-023-01328-x (PMC9867758; doi:10.1038/s41420-023-01328-x)

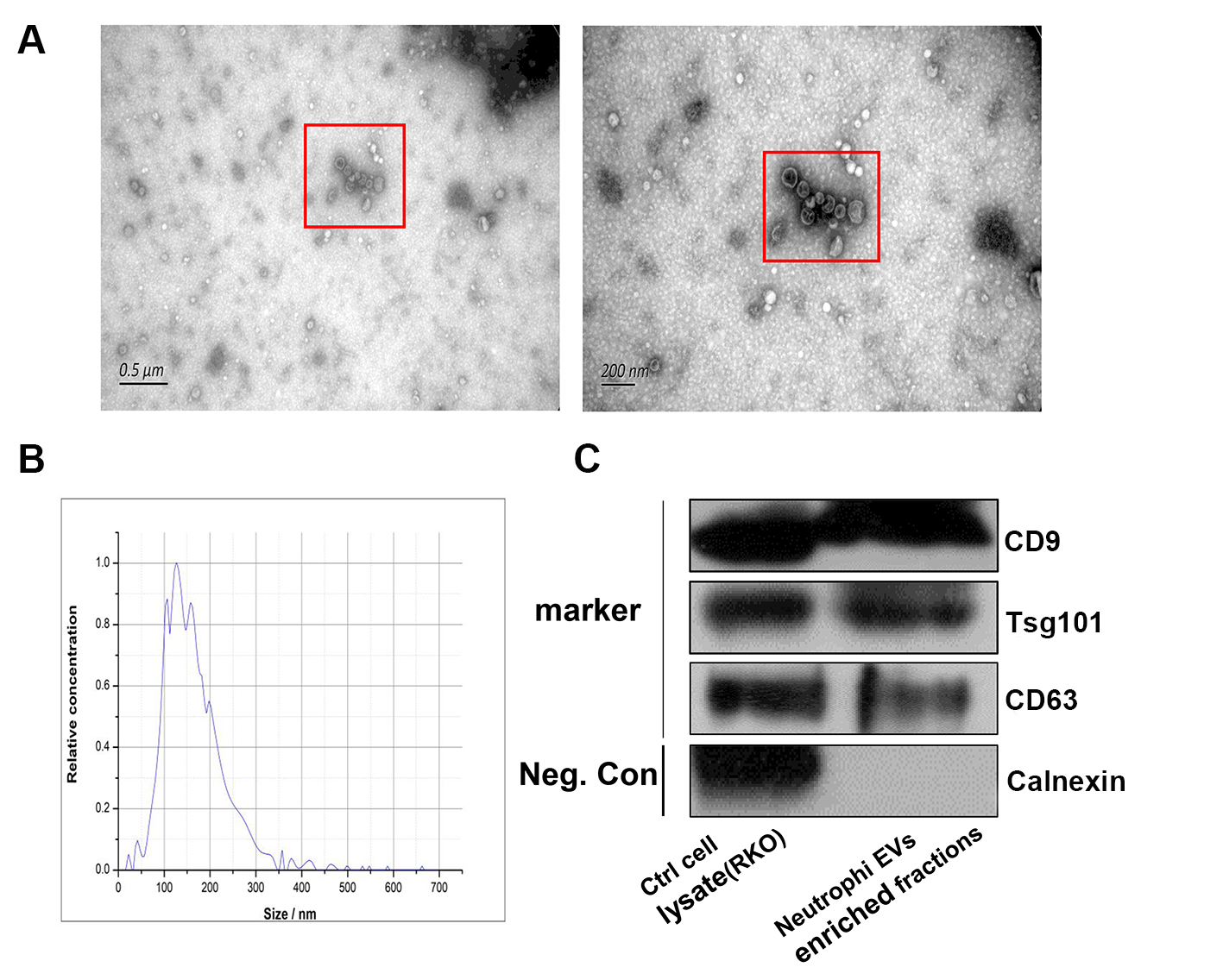

Supplement: Supplementary file 7 — Figure S1 [file 41420_2023_1328_MOESM7_ESM.tif]

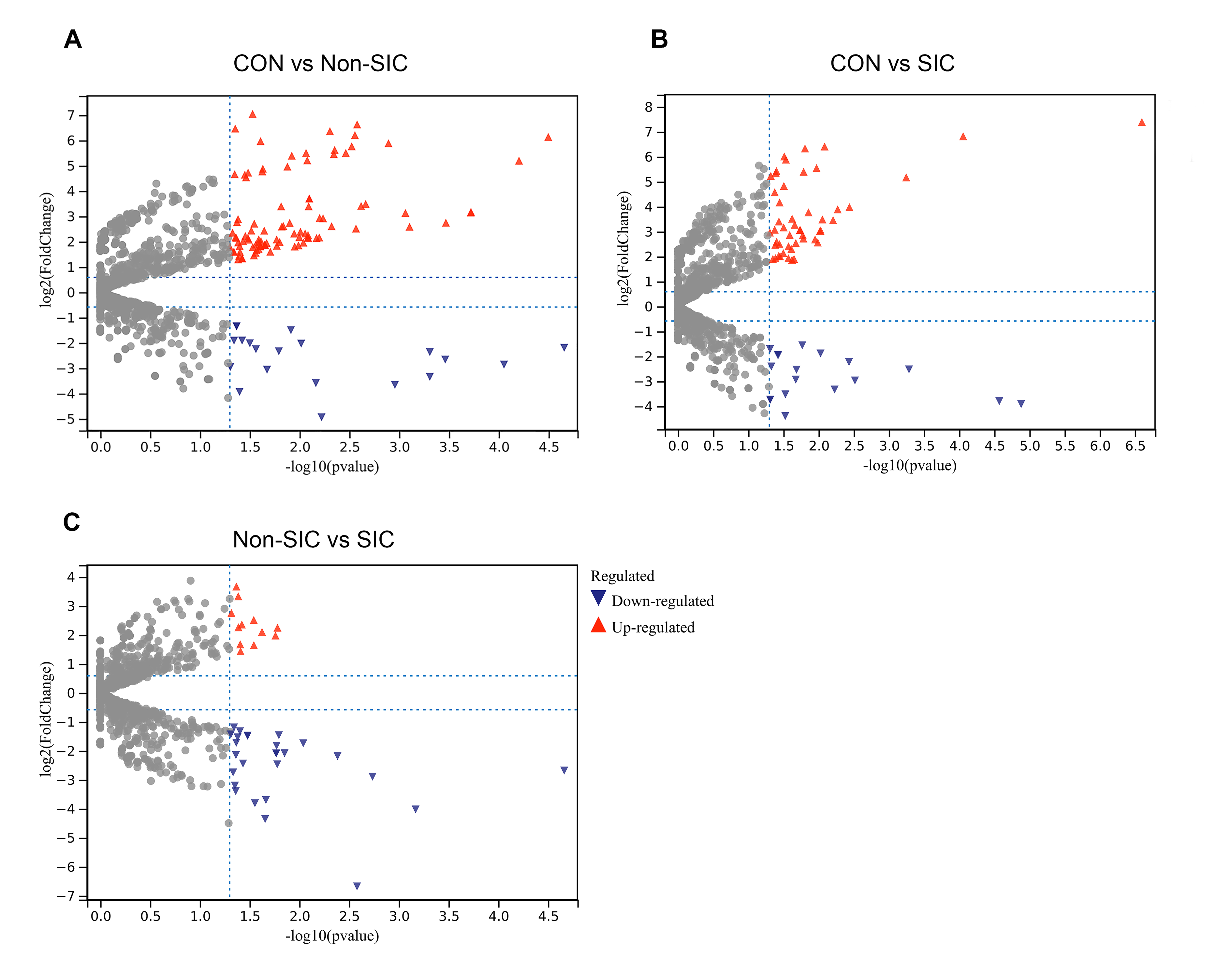

Supplement: Supplementary file 8 — Figure S2 [file 41420_2023_1328_MOESM8_ESM.tif]

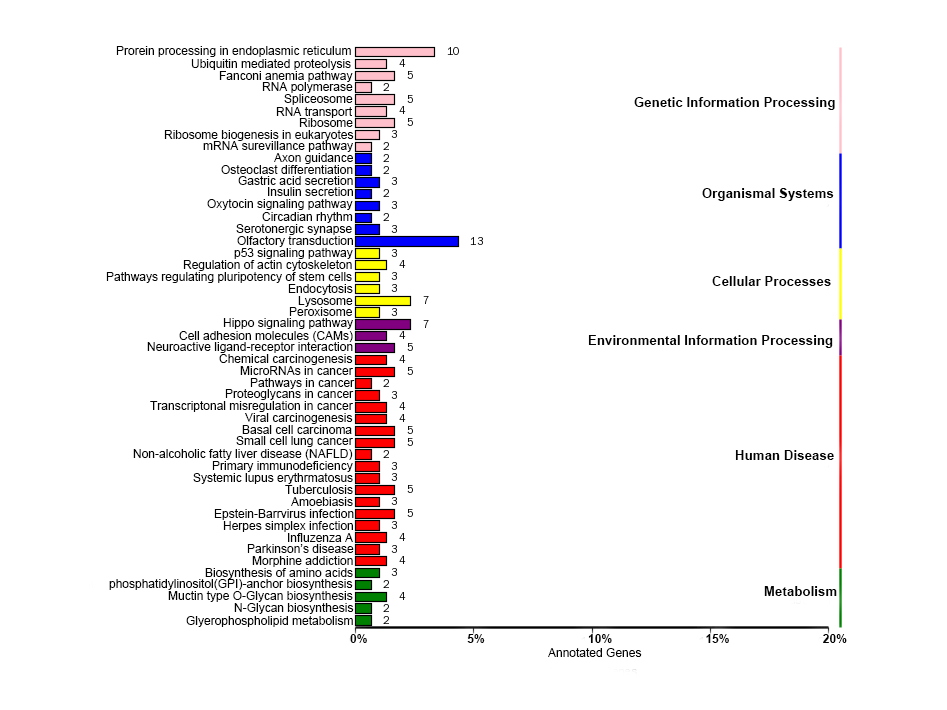

Supplement: Supplementary file 9 — Figure S3 [file 41420_2023_1328_MOESM9_ESM.tif]

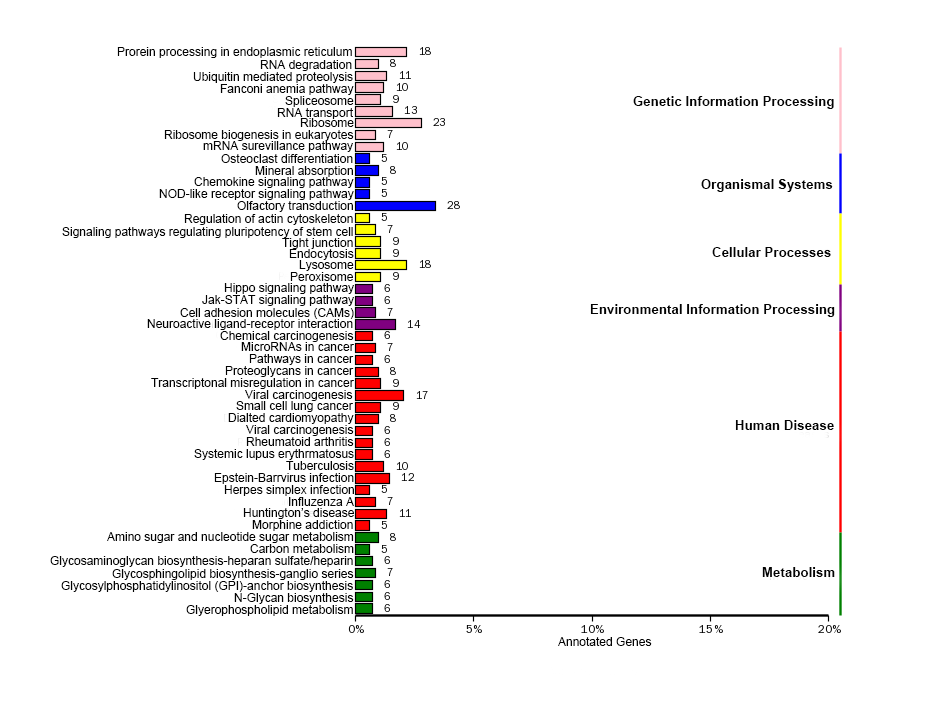

Supplement: Supplementary file 10 — Figure S4 [file 41420_2023_1328_MOESM10_ESM.tif]

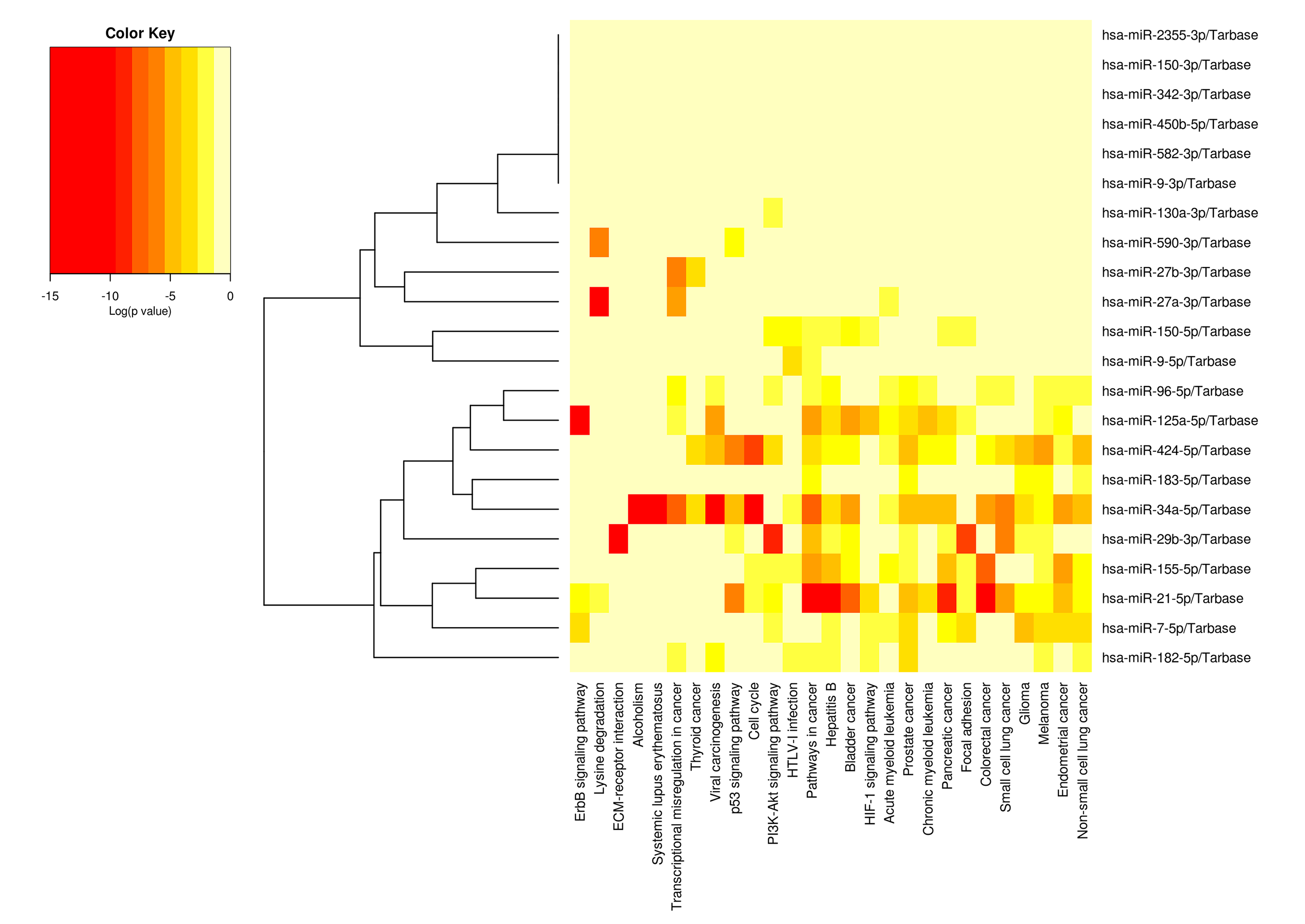

Supplement: Supplementary file 11 — Figure S5 [file 41420_2023_1328_MOESM11_ESM.tif]

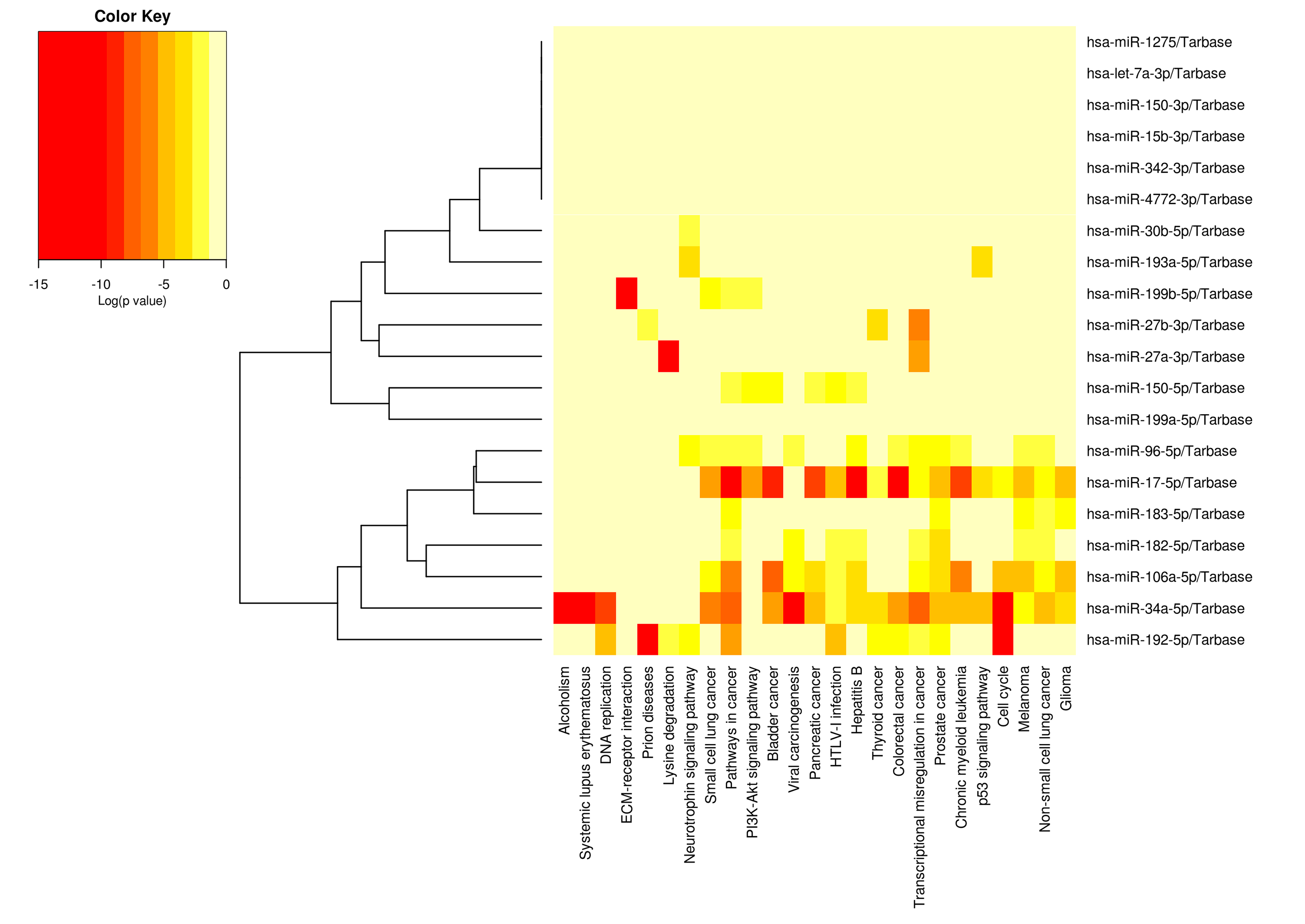

Supplement: Supplementary file 12 — Figure S6 [file 41420_2023_1328_MOESM12_ESM.tif]

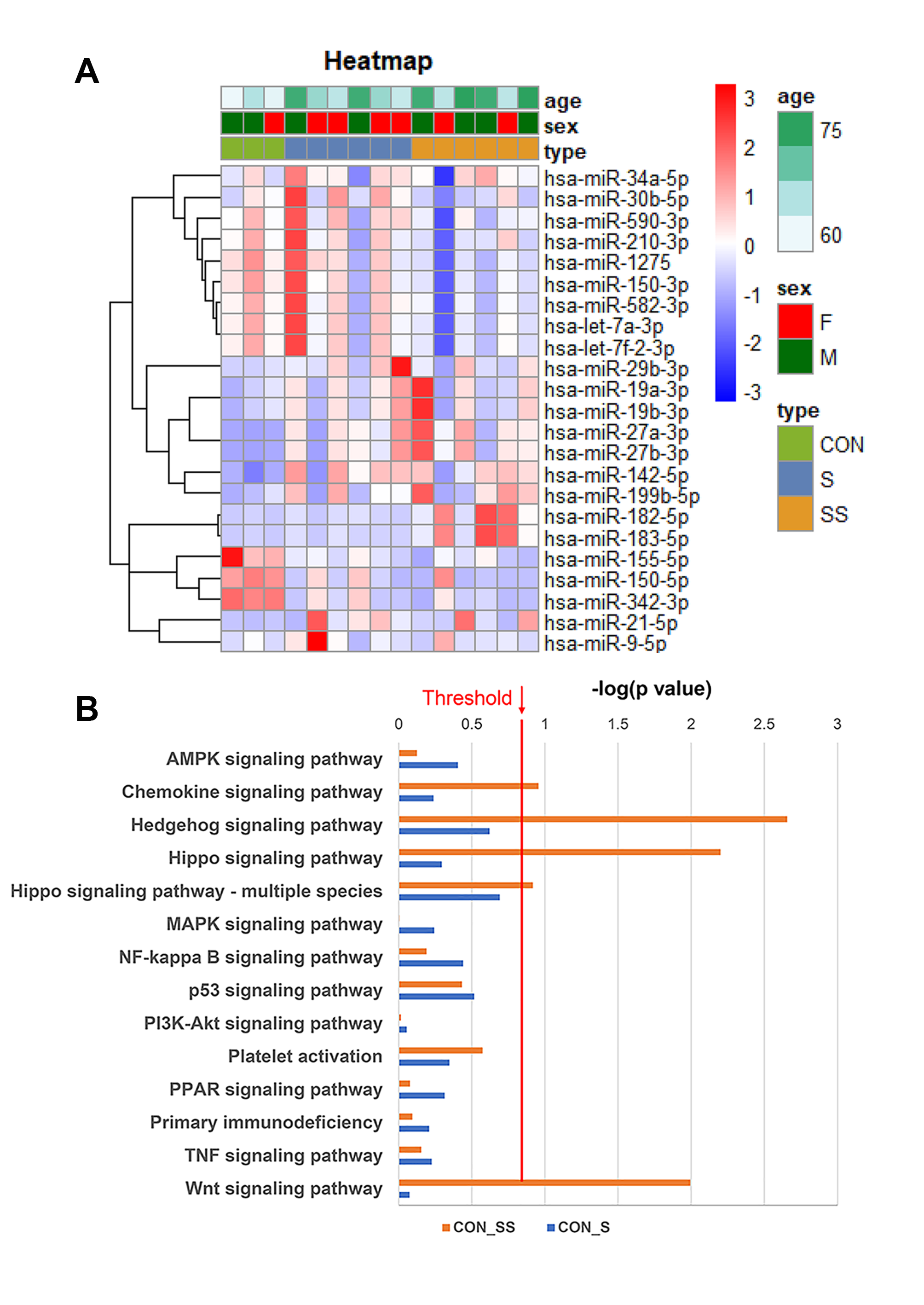

Supplement: Supplementary file 13 — Figure S7 [file 41420_2023_1328_MOESM13_ESM.tif]

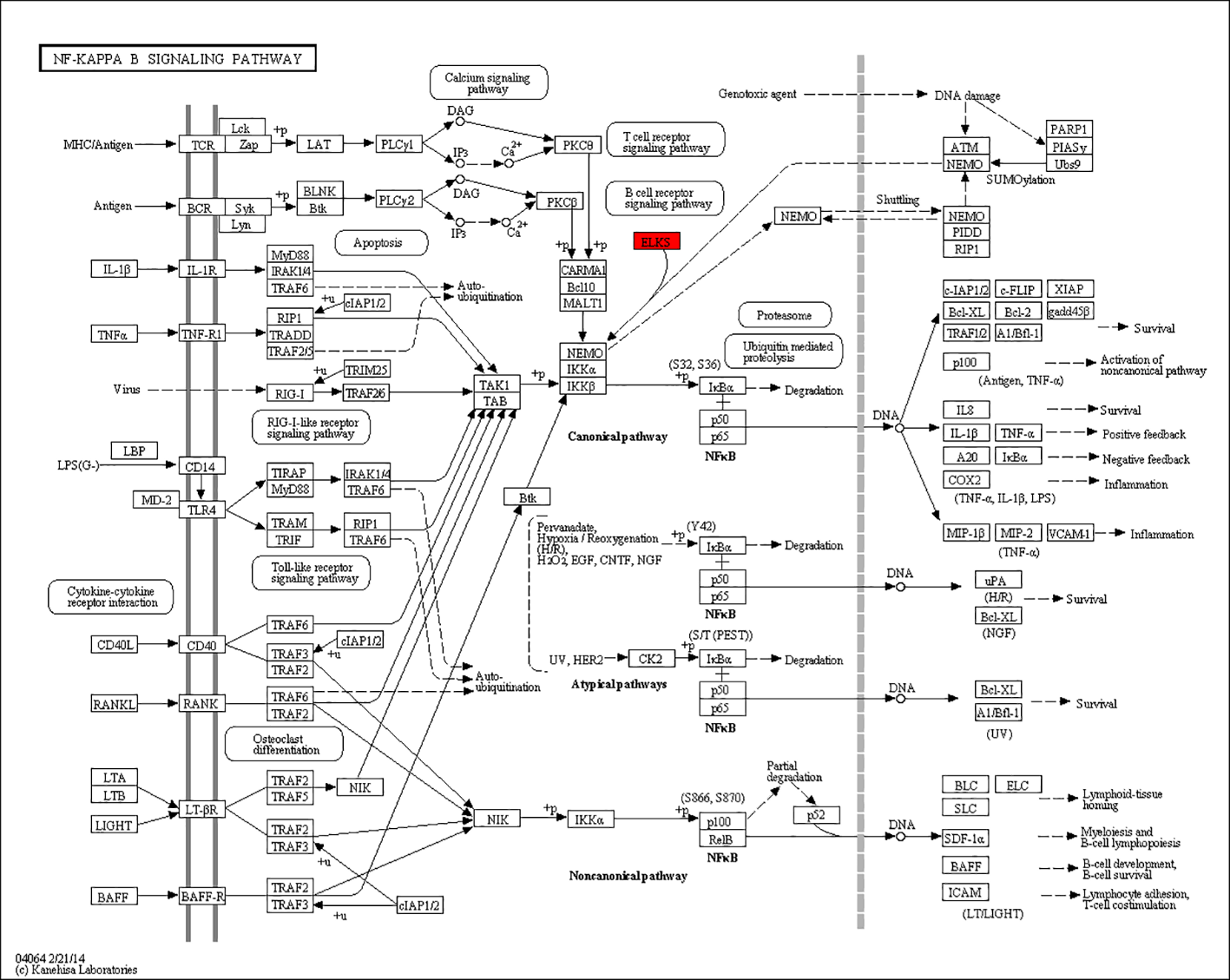

Supplement: Supplementary file 14 — Figure S8 [file 41420_2023_1328_MOESM14_ESM.tif]

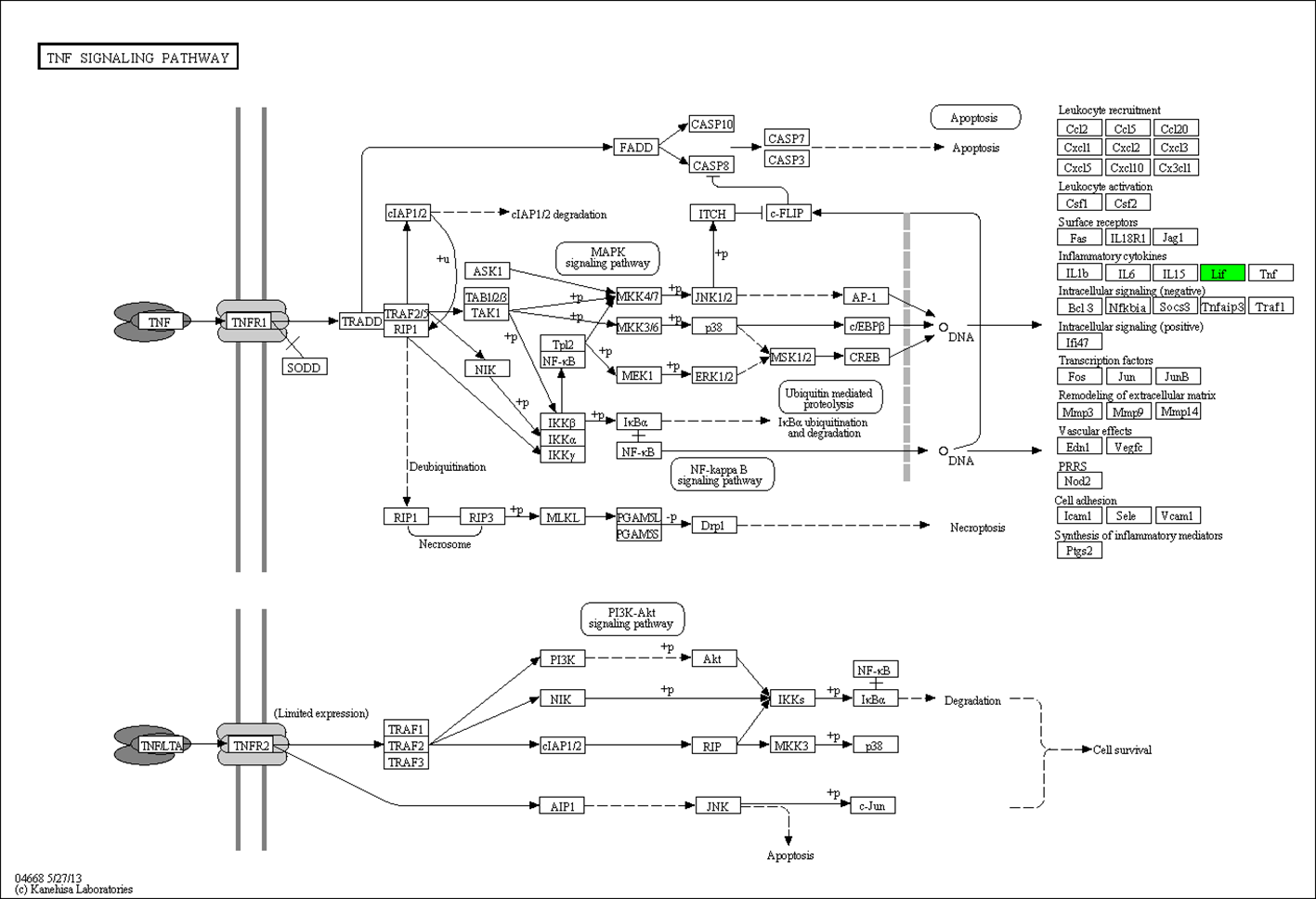

Supplement: Supplementary file 15 — Figure S9 [file 41420_2023_1328_MOESM15_ESM.tif]

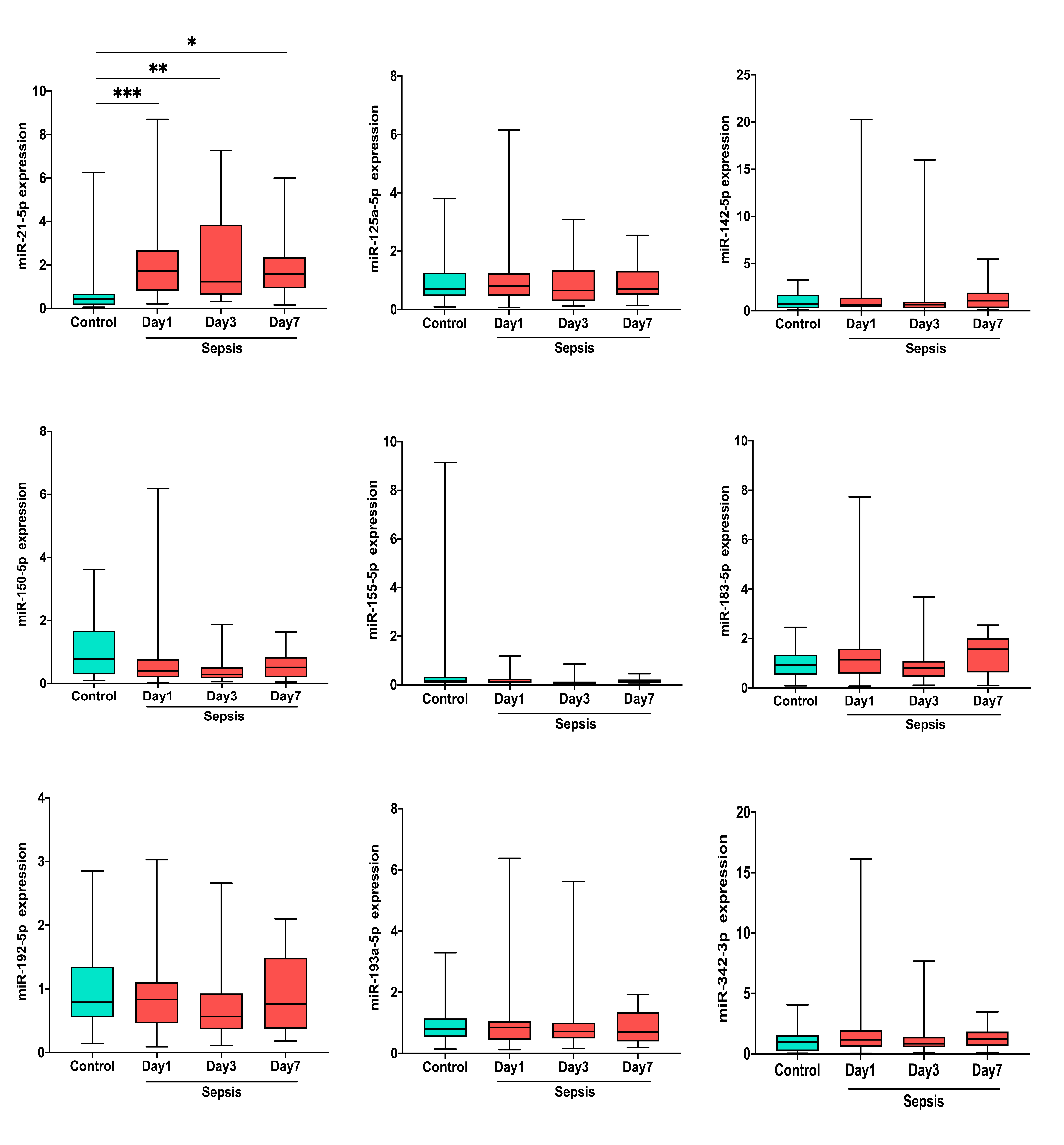

Supplement: Supplementary file 16 — Figure S10 [file 41420_2023_1328_MOESM16_ESM.tif]

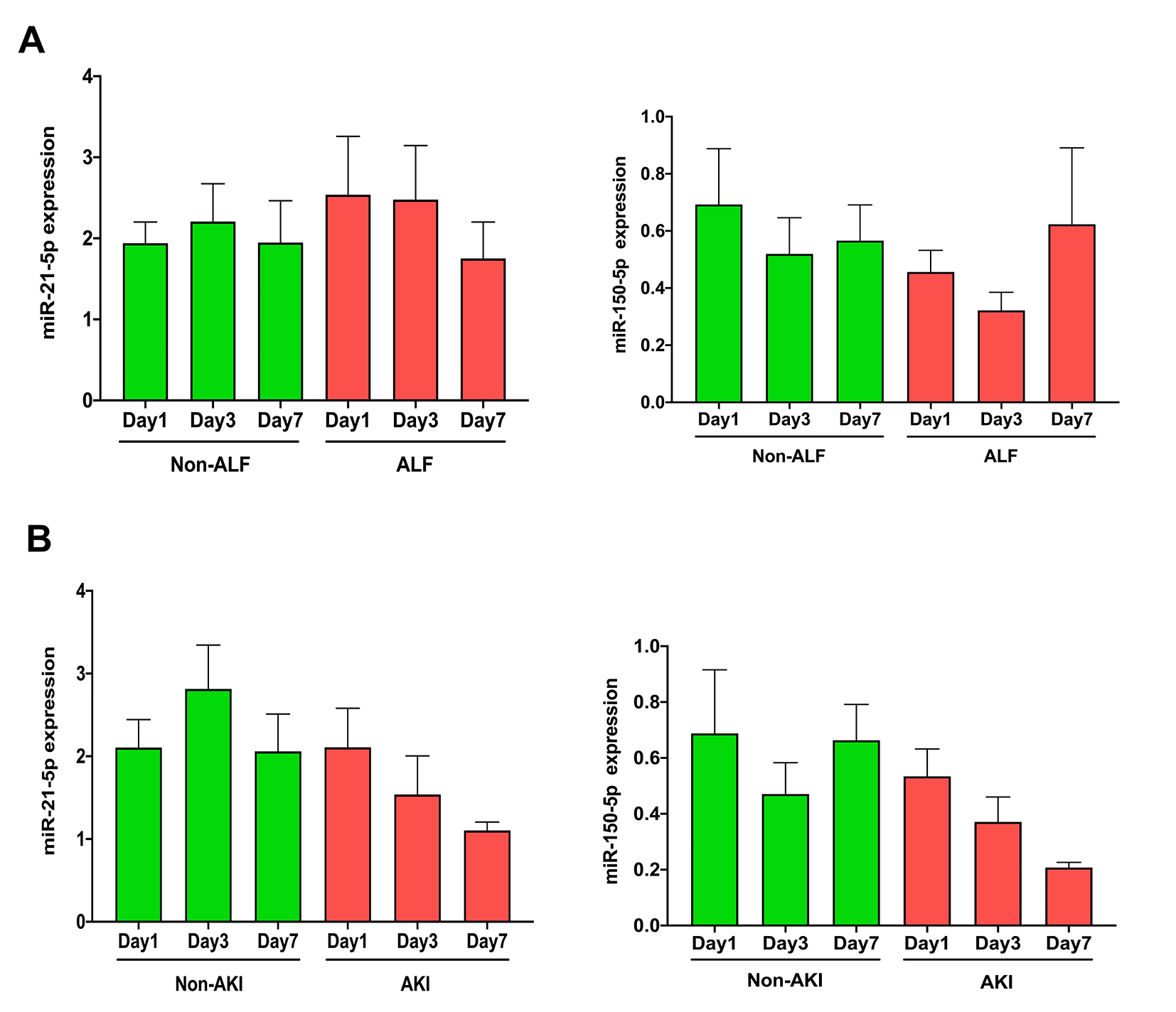

Supplement: Supplementary file 17 — Figure S11 [file 41420_2023_1328_MOESM17_ESM.tif]

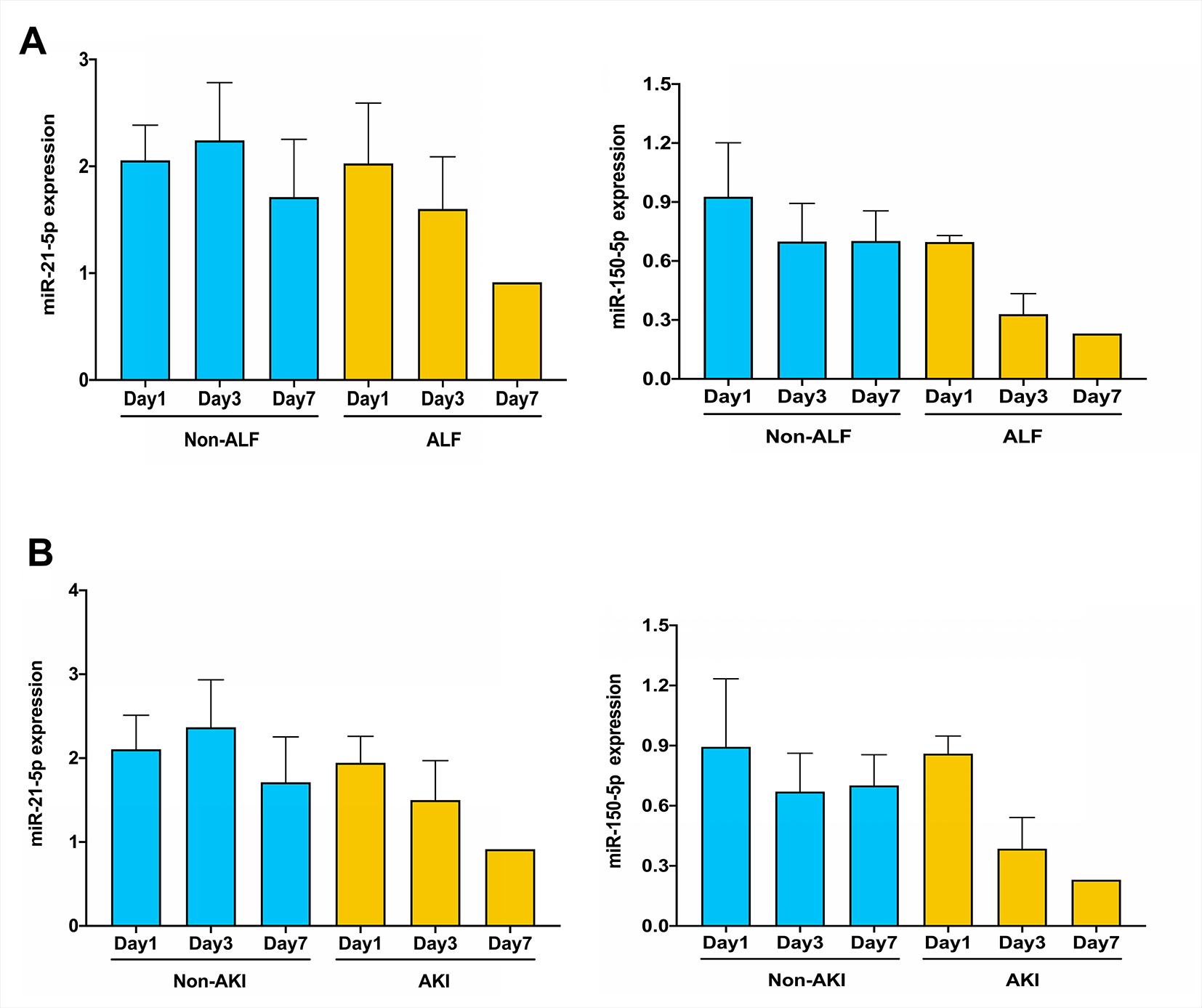

Supplement: Supplementary file 18 — Figure S12 [file 41420_2023_1328_MOESM18_ESM.tif]

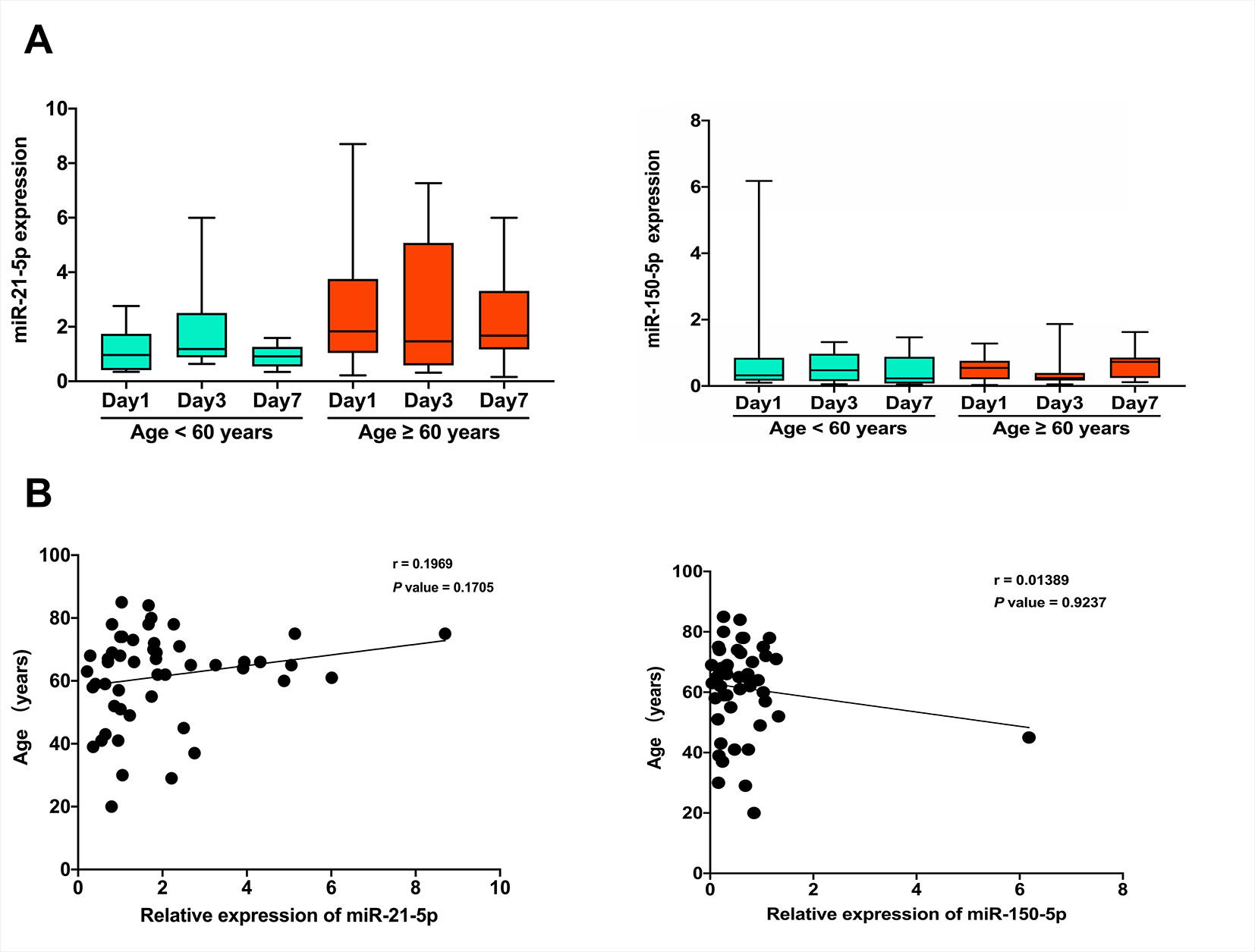

Supplement: Supplementary file 19 — Figure S13 [file 41420_2023_1328_MOESM19_ESM.tif]

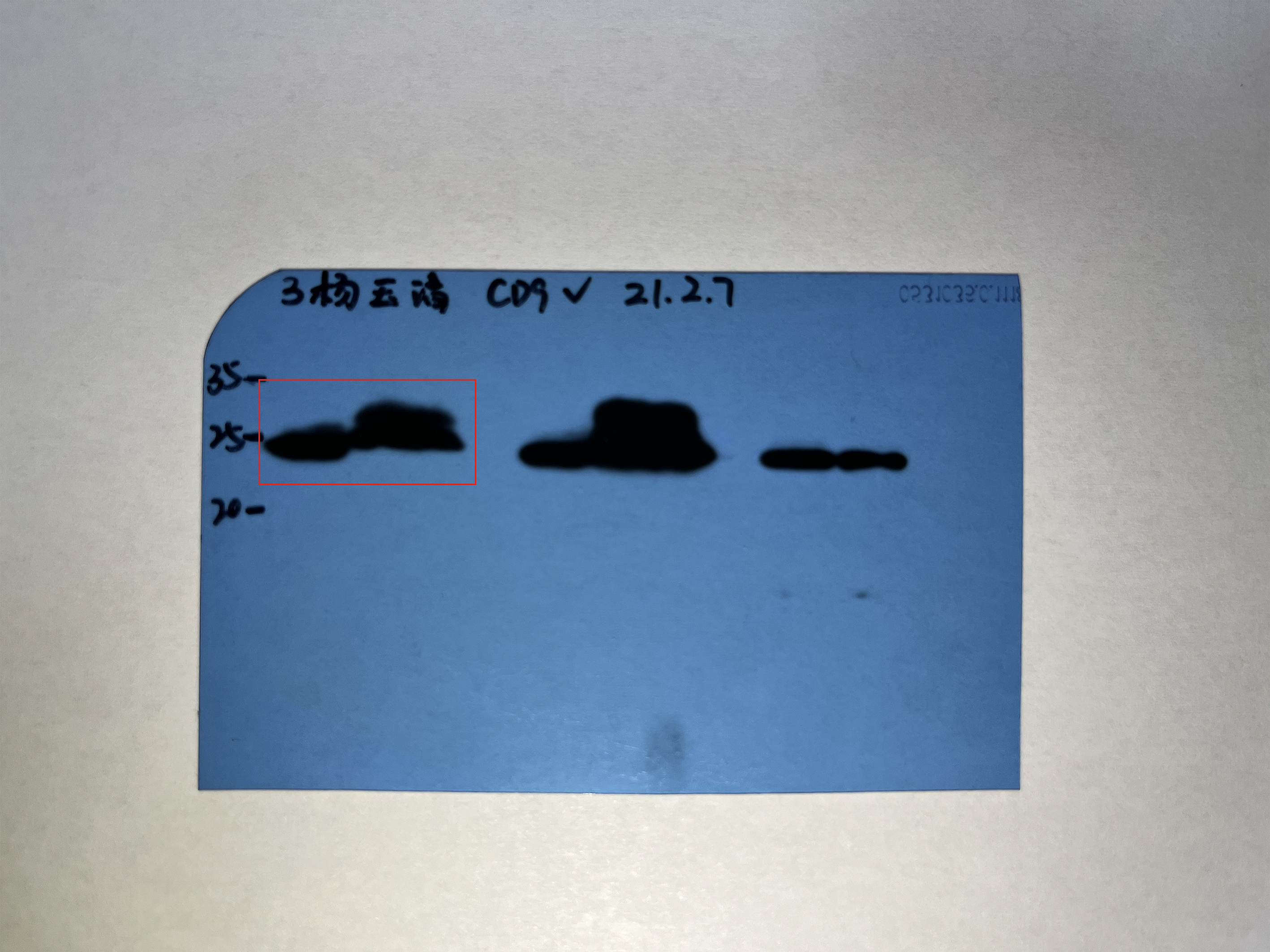

Supplement: Supplementary file 20 — Original Data File [file 41420_2023_1328_MOESM20_ESM.tif]

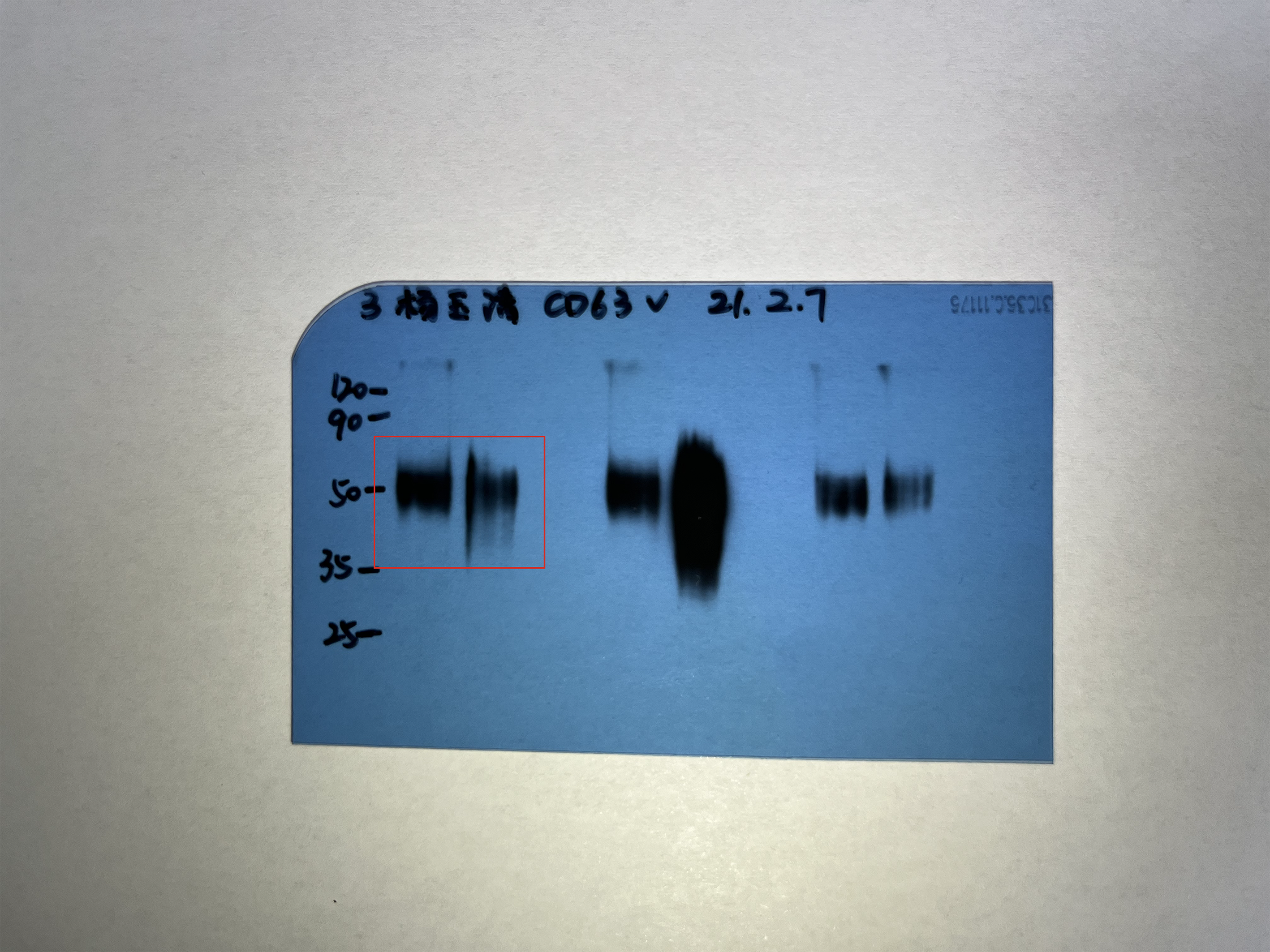

Supplement: Supplementary file 21 — Original Data File [file 41420_2023_1328_MOESM21_ESM.tif]

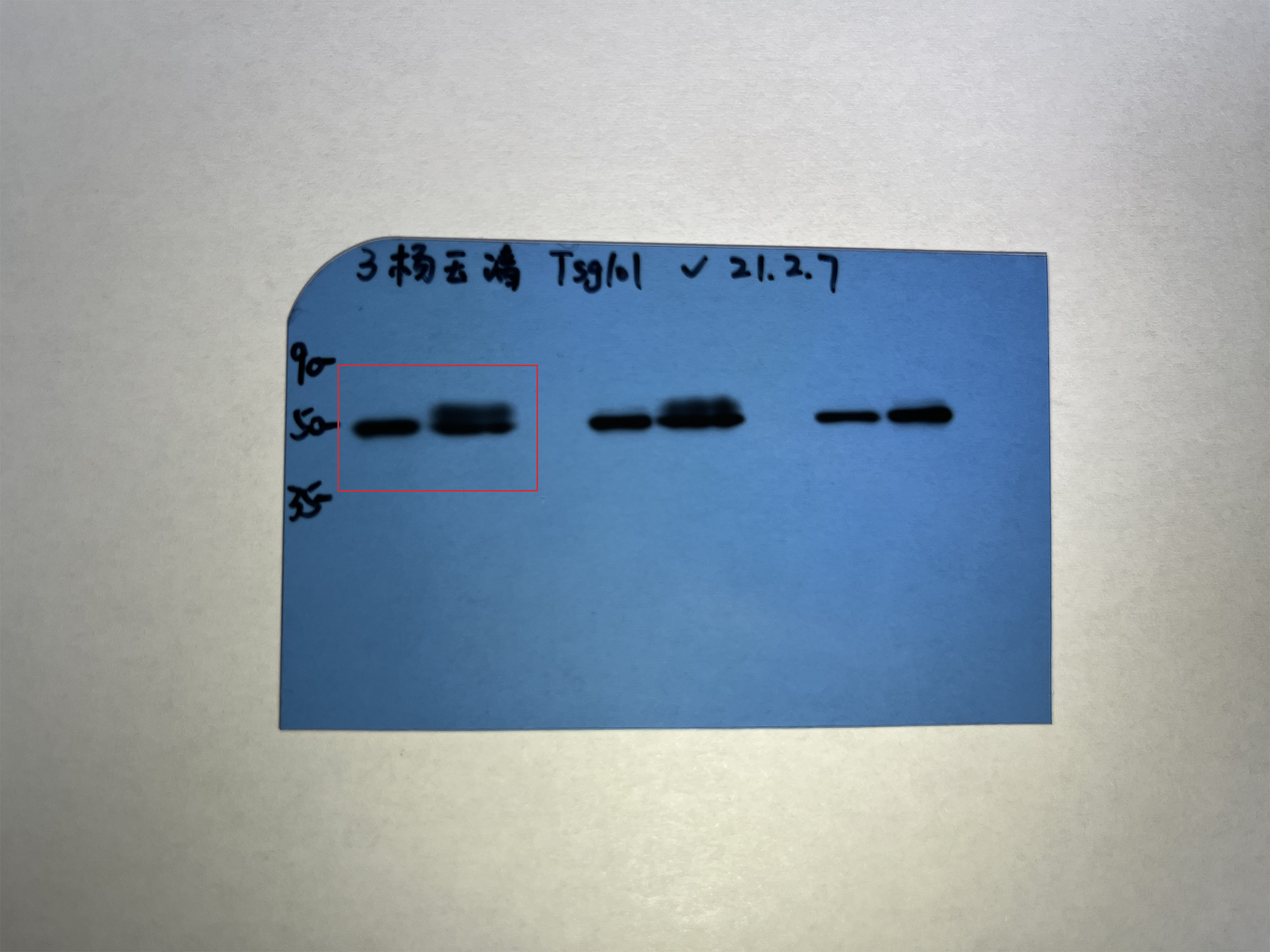

Supplement: Supplementary file 22 — Original Data File [file 41420_2023_1328_MOESM22_ESM.tif]

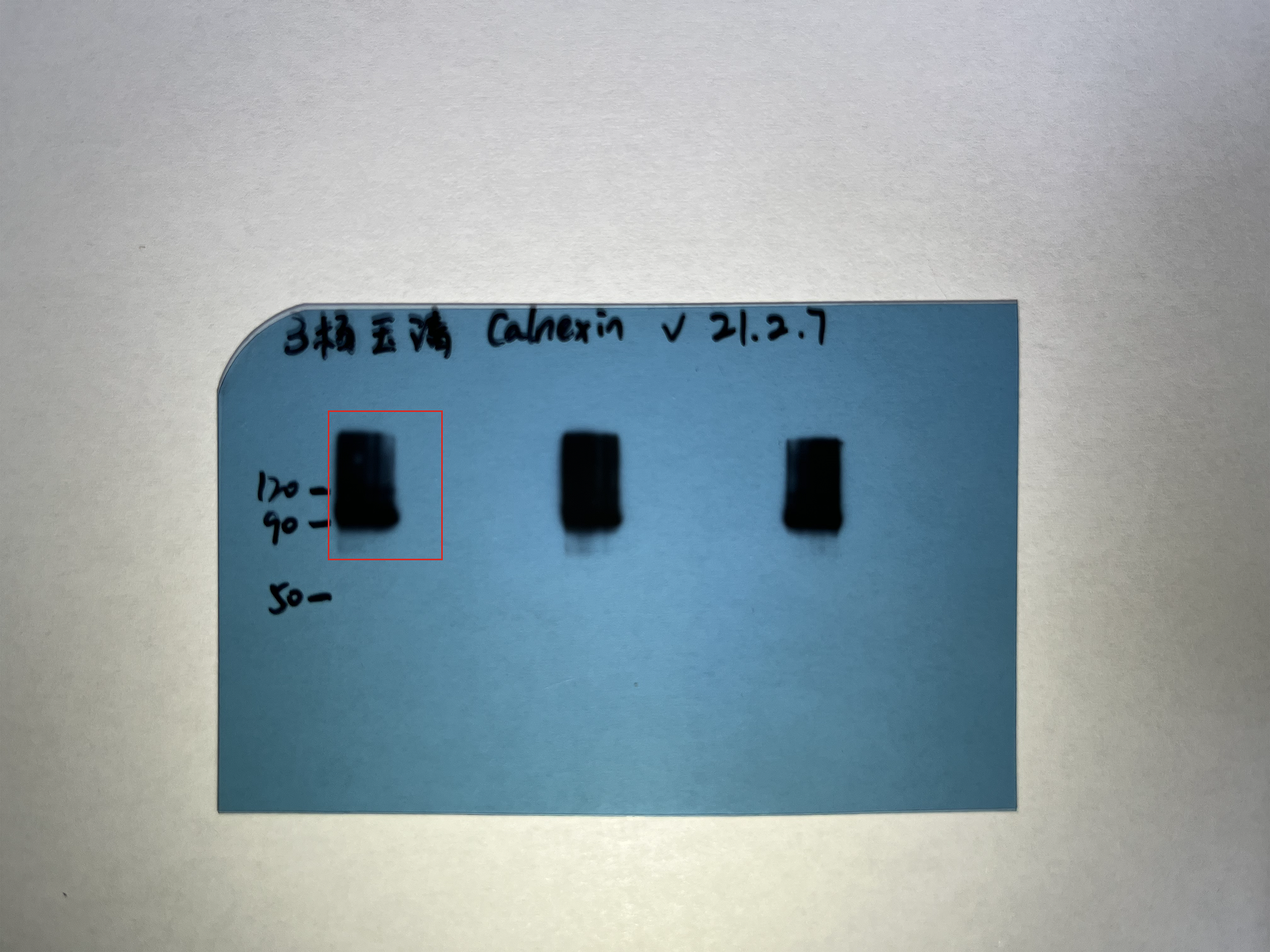

Supplement: Supplementary file 23 — Original Data File [file 41420_2023_1328_MOESM23_ESM.tif]
